# Remote, aerial phenotyping of maize traits with a mobile multi-sensor approach

**DOI:** 10.1186/s13007-015-0048-8

**Published:** 2015-02-25

**Authors:** Frank Liebisch, Norbert Kirchgessner, David Schneider, Achim Walter, Andreas Hund

**Affiliations:** Institute of Agricultural Sciences, ETH Zürich, Universitätstrasse 2, 8092 Zürich, Switzerland; Norddeutsche Pflanzenzucht, Hohenlieth, Holtsee D-24363 Germany

**Keywords:** Remote sensing, Aerial phenotyping, Near infrared imaging, Image analysis, NDVI, Thermal imaging, *Zea mays*

## Abstract

**Background:**

Field-based high throughput phenotyping is a bottleneck for crop breeding research. We present a novel method for repeated remote phenotyping of maize genotypes using the Zeppelin NT aircraft as an experimental sensor platform. The system has the advantage of a low altitude and cruising speed compared to many drones or airplanes, thus enhancing image resolution while reducing blurring effects. Additionally there was no restriction in sensor weight. Using the platform, red, green and blue colour space (RGB), normalized difference vegetation index (NDVI) and thermal images were acquired throughout the growing season and compared with traits measured on the ground. Ground control points were used to co-register the images and to overlay them with a plot map.

**Results:**

NDVI images were better suited than RGB images to segment plants from soil background leading to two separate traits: the canopy cover (CC) and its NDVI value (NDVI_Plant_). Remotely sensed CC correlated well with plant density, early vigour, leaf size, and radiation interception. NDVI_Plant_ was less well related to ground truth data. However, it related well to the vigour rating, leaf area index (LAI) and leaf biomass around flowering and to very late senescence rating. Unexpectedly, NDVI_Plant_ correlated negatively with chlorophyll meter measurements. This could be explained, at least partially, by methodical differences between the used devices and effects imposed by the population structure. Thermal images revealed information about the combination of radiation interception, early vigour, biomass, plant height and LAI. Based on repeatability values, we consider two row plots as best choice to balance between precision and available field space. However, for thermography, more than two rows improve the precision.

**Conclusions:**

We made important steps towards automated processing of remotely sensed data, and demonstrated the value of several procedural steps, facilitating the application in plant genetics and breeding. Important developments are: the ability to monitor throughout the season, robust image segmentation and the identification of individual plots in images from different sensor types at different dates. Remaining bottlenecks are: sufficient ground resolution, particularly for thermal imaging, as well as a deeper understanding of the relatedness of remotely sensed data and basic crop characteristics.

**Electronic supplementary material:**

The online version of this article (doi:10.1186/s13007-015-0048-8) contains supplementary material, which is available to authorized users.

## Background

Field-based high-throughput phenotyping methods are urgently needed by plant breeding research [1,2]. Whereas laboratory-based phenotyping platforms that monitor the performance of single plants of model species have advanced greatly in recent years (e.g. [3], for a review see [4]), the development of field-based phenotyping approaches has lagged. For field-based methods, progress has been made mostly using camera-based approaches that are mounted on ground-based vehicles like tractors (e.g. [5,6]; for a review see [2,7]). Yet, there is little progress on methods and platforms that operate from the air [1] although currently drones are becoming increasingly popular for aerial photography. However, high quality camera systems often still exceed the payload of available drones. Automation of data processing, difficulties in extraction of meaningful parameters and blurry images taken from conventional carrier systems such as airplanes travelling at relatively high altitude are other reasons which presently restrict fast methodological advances. Nevertheless, the potential throughput of airborne phenotyping approaches is intrinsically higher than that of ground-based approaches, for several reasons: (1) wider viewing angle from the air, (2) potentially higher travelling speed, (3) absence of physical contact with and hence no mechanical distraction of the growing crop and (4) independence of wet soil conditions that prevent traffic on the ground.

Maize is one of the most important staple crops and has gained an enormous importance in tropical and temperate regions as a food, fodder, and energy crop. As a consequence there is a high need to develop high-throughput methods for hybrid breeding of maize in order to increase selection efficiency [8-11]. Relevant breeding approaches require field-based testing of their genotypes [12]. Often hundreds or thousands of genotypes need to be investigated for their performance in the field and hence need to be grown and assessed synchronously side by side. It is widely accepted that in such breeding programs, phenotyping of traits that are related to yield and quality is currently constituting a serious bottleneck [2,13], for which the development of technological possibilities has not kept pace with the genomic characterization of the germplasm.

Therefore, we aimed to develop a concept allowing for 1) continuous measurements of genotypes throughout the growing season using RGB and near infrared imaging and thermography, 2) develop protocols to automatically identify individual field plots in images derived by the different sensors at different dates and from slightly different angles, 3) identify suitable traits and optimal plot size based on the repeatability and 4) relate remotely sensed data to ground truth data.

**Figure 1:**
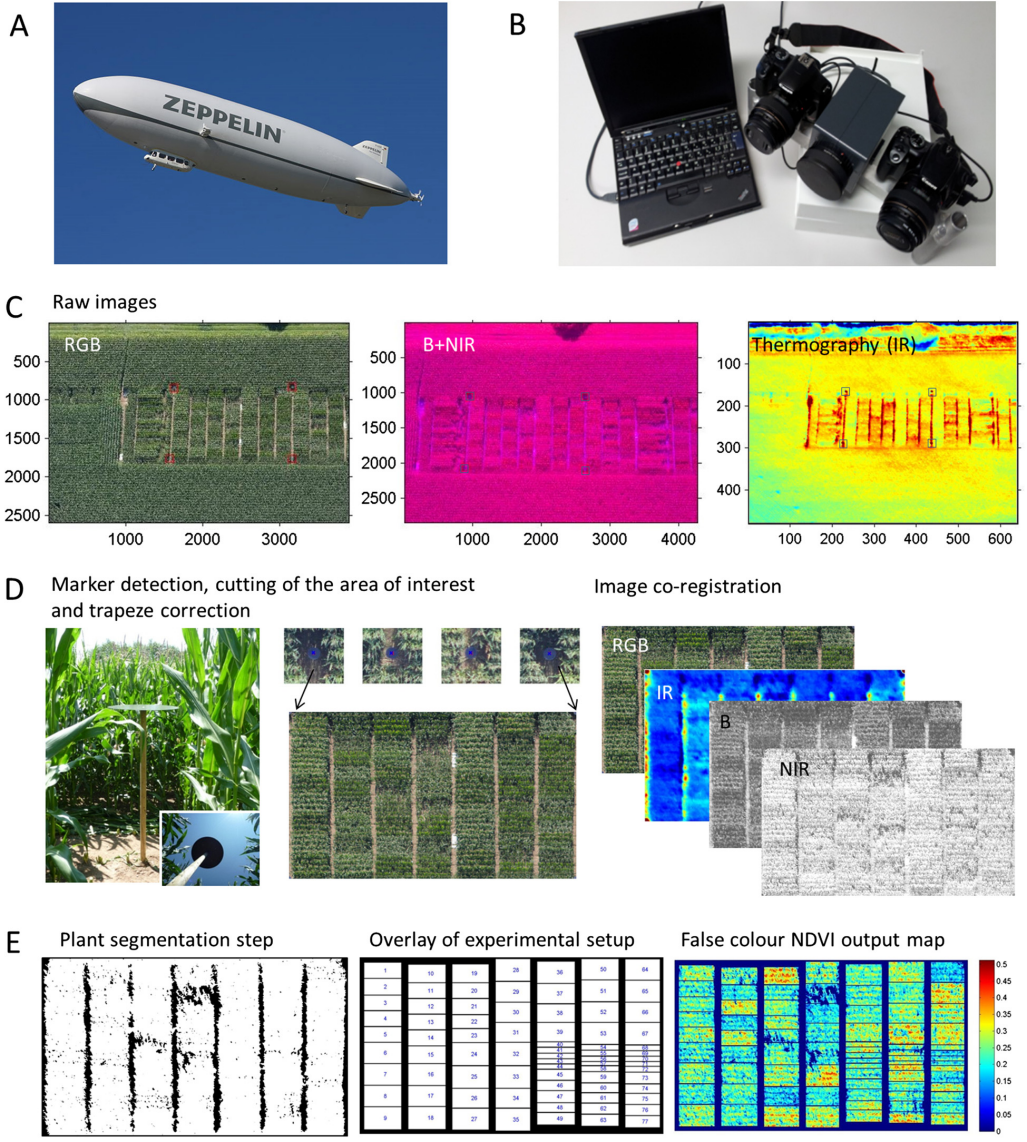
**Illustration of the imaging and analysis pipeline. (A)** Aerial platform Zeppelin NT, **(B)** handheld sensor array consisting of three cameras, **(C)** images derived by: a consumer camera (RGB), a modified consumer camera for vegetation detection (B + NIR) and an infrared camera (Thermography, IR). Squares indicate location of field markers. **(D)** Round black metal plates serve as field markers (left) for automatic detection and subsequent clipping of the area of interest (AoI) and trapeze correction of the raw images (middle). Co-registered tiff images for the RGB composite and the three data channels IR, B and NIR (right). **(E)** Three steps of image procession: mask to segment plant from soil pixel (left), mask to identify plots (middle) and a combined output map of NDVI_Plant_ values within plots in false colour (right). The shown images represent one of three parts of the field separated for measurement purposes, details described in the material section. See Additional file 1 for a complete image of the experiment.

More specifically, this study investigates the application of a camera combination consisting of (1) a standard RGB camera, (2) a camera to determine the normalized difference vegetation index (NDVI) and (3) a high-resolution thermal camera (Table 1). This sensor array was operated manually on a Zeppelin aircraft offering regular sight-seeing round trips. The maize experiment was placed on one of the flight tracks in order to ensure frequent monitoring during the growing season (Figure 1). The experimental field contained 16 different maize genotypes, arranged in a well-designed plot structure with plots of multiple sizes (i.e. different number of rows). Each genotype x plot size combination was replicated four times (Additional file 1). From the acquired images, parameters such as the canopy cover, leaf greenness and canopy temperature were detected, and a software routine was developed that allowed for (semi-) automated identification of and data extraction from the field plot structure. The extracted parameter values were then correlated with ground measurements of relevant crop traits collected throughout the crop development. They comprise phenological traits (like the time needed to reach certain key developmental stages) and morphological characteristics (like plant height and leaf biomass) that contribute to the performance of a genotype in a given environment. We hypothesize that the elaborated methods of image capture and analysis can be used to identify genotypic differences and changes during development of maize throughout the season and that remotely sensed parameters can be related sufficiently well to ground measured plant properties and traits relevant for breeding.

**Table 1:** **Size of ground images (length and width) and effective pixel dimensions (Instantaneous Field of View = IFoV) as affected by sensor resolution and measurement altitude**

**Camera**	**Lens (focal length)**	**Sensor resolution**	**Sensor dimensions**	**Image parameter**	**Altitude 290**	**300**	**310**
	**mm**	**pixel**	**mm**		**m**	**m**	**m**
NIR	60	4282 × 2848	22.2 × 14.8	length	107.6	111.3	115.5
				width	71.5	74.0	76.5
				IFoV^a^	0.025	0.026	0.028
RGB	60	3898 × 2595	22.2 × 14.8	length	107.3	111.1	114.7
				width	71.4	73.9	76.4
				IFoV	0.0275	0.0285	0.0295
IR	75	640 × 480	14.9 × 11.2^b^	length	61.9	64	66.1
				width	46.4	48	49.6
				IFoV	0.096	0.1	0.103

^a^IFoV = (pixel dimension*distance)/focal length.

^b^IFoV = (sensor pixel size* distance to ground)/focal length, derived from companies IFoV calculator.

## Results

### Processing of image-based signals

A semi-automated recognition of black metal markers on 2 m high poles worked well for all sensor outputs. The markers were distinguishable from the soil and plant signal by all three cameras (Figure 1). However, cleaning of the field markers before flight campaigns was necessary, especially during pollen shedding. White tarps that were put on the ground were less useful as markers since they were easily covered with soil, particularly after rains. Cleaning of the white tarps proved to be too laborious and time consuming. Moreover, with increasing plant size the white tarps were progressively obscured by the maize plants, changing the detectable marker shape and increasing the need for manual co-registration of the images. However, the tarps were very useful as landmarks for the pilots when the plants were small, and the plots were difficult to detect.

After identification in the images, the black metal markers were used to clip the images to the area of interest (AoI) and subsequently co-register all sensor output images and correct them for trapeze distortion (Figure 1). On top of the aligned and corrected image stacks, the prepared experimental plot mask was projected for subsequent plotwise data extraction.

**Figure 2:**
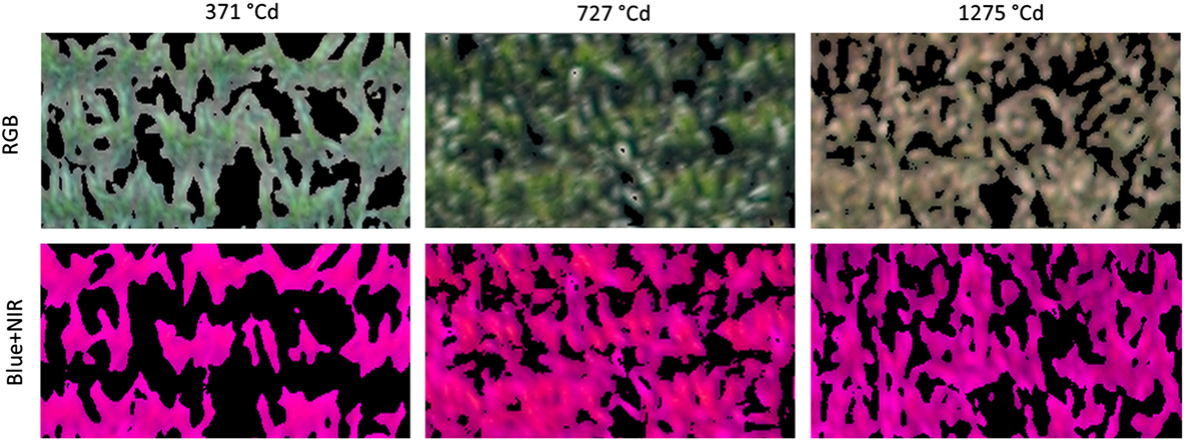
**Comparison of HSB and NDVI based threshold segmentation masks laid over unprocessed RGB and B + NIR images, respectively, when the same colour or NDVI thresholds were applied throughout the season (see materials section for further info).**

The most basic information derived from the images of the vegetation camera, was the plot-based NDVI (NDVI_Plot_) including both, plant and soil information. A segmentation process based on the NDVI or RGB information led to two additional traits: the canopy cover (CC) and the NDVI values of the area covered by plants (NDVI_Plant_). CC was best calculated from NDVI images since throughout the season the same segmentation threshold could be used whereas for RGB images, it had to be adjusted for each measurement campaign (Figure 2). For NDVI images, only maximal signal intensity needed to be adjusted to comparable conditions for the different measurement campaigns. Using a NDVI threshold of 0.1 excluded non-plant material and included green and senescent plant material. A threshold of 0.2 excluded large parts of the senescent plant area as well (data not shown).

### Seasonal development of canopy cover was most reliably evaluated in plots with more than two rows

**Figure 3:**
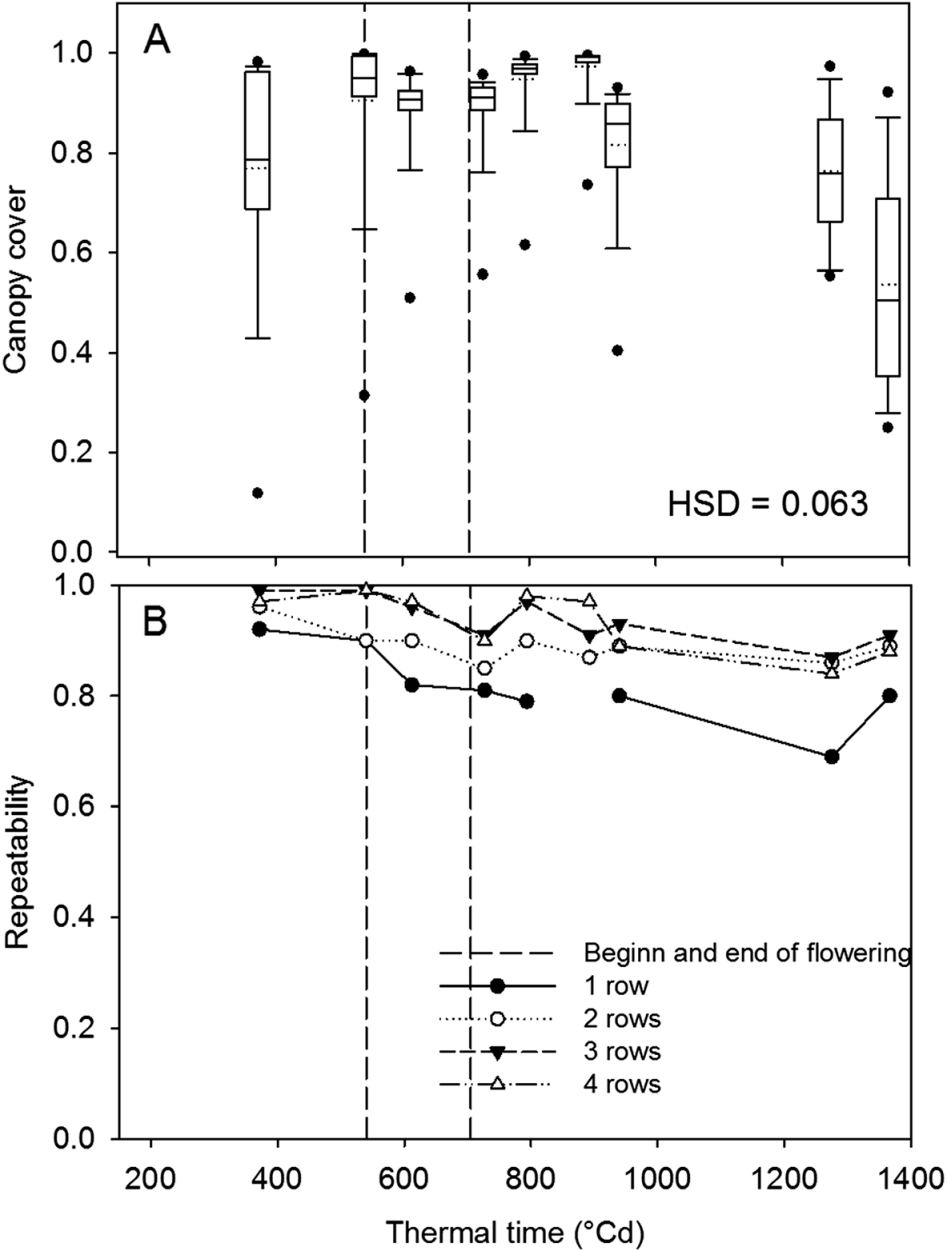
**Canopy cover of four row plots (A) and repeatability of canopy cover affected by plot size (B).** Boxplots are based on mean values of the 16 genotypes. The solid line in the box indicates the median and the dotted line the mean.

The canopy cover increased until flowering (540°Cd) and decreased during the late senescence phase (after 892°Cd; Figure 3). The corresponding dates and growth stages can be found in Table 2. The canopies of nearly all investigated genotypes were closed at the onset of flowering indicated by CC values above 0.95. At this stage, only genotypes 6 and 15 showed CC below 0.9 (data not shown). A small reduction of CC was observed during flowering and shortly after flowering between 540 and 793°Cd (Figure 3) and likely was related to green leaf area overlayed by tassels and anthers.

**Table 2:** **Dates of aerial image acquisition**

**Flight**	**Date** ^**a**^	**Time** ^**b**^	**DAS** ^**c**^	**TT** ^**d**^	**Camera line up**	**Growth stage**
1	16.06.2011	8:38	56	371	NIR, RGB	leaf 6 to 9 fully developed
2	05.07.2011	17:18	75	540	NIR, RGB	Begin of tasseling
3	11.07.2011	17:47	81	612	NIR, RGB, IR	Most genotypes tasseling (>50%)
4	26.07.2011	16:43	96	727	NIR, RGB, IR	All genotypes tasseling (100%)
5	02.08.2011	17:22	103	793	NIR, RGB, IR	Begin of corn filling
6	12.08.2011	17:22	113	893	NIR, RGB, IR	Begin of leaf senescence
7	16.08.2011	17:26	117	940	NIR, RGB, IR	
8	15.09.2011	14:30	147	1275	NIR, RGB, IR	Late senescence, upper leaf levels affected
9	29.09.2011	17:47	161	1366	NIR, RGB	Full maturity of most genotypes (black layer observed)

^a^day. month. year, ^b^Central European time, ^c^days after sowing, ^d^thermal time (in °Cd).

To elucidate which plot size (row number) was sufficient to differentiate among genotypes we used the repeatability, i.e. the proportion of the genotypic variation compared to the overall phenotypic variation. The repeatability of CC was above 0.95 before onset of tassels for the three and four row plots decreasing slightly to 0.85 at maturity. As expected, the smaller plot size showed lower repeatability, especially for the one row plots. There, values ranged from 0.9 before flowering down to 0.7 close to maturity. Clearly, three- to four row plots were preferable for this type of aerial observations.

### Seasonal development of NDVI was most reliably measured in plots with more than one row

**Figure 4:**
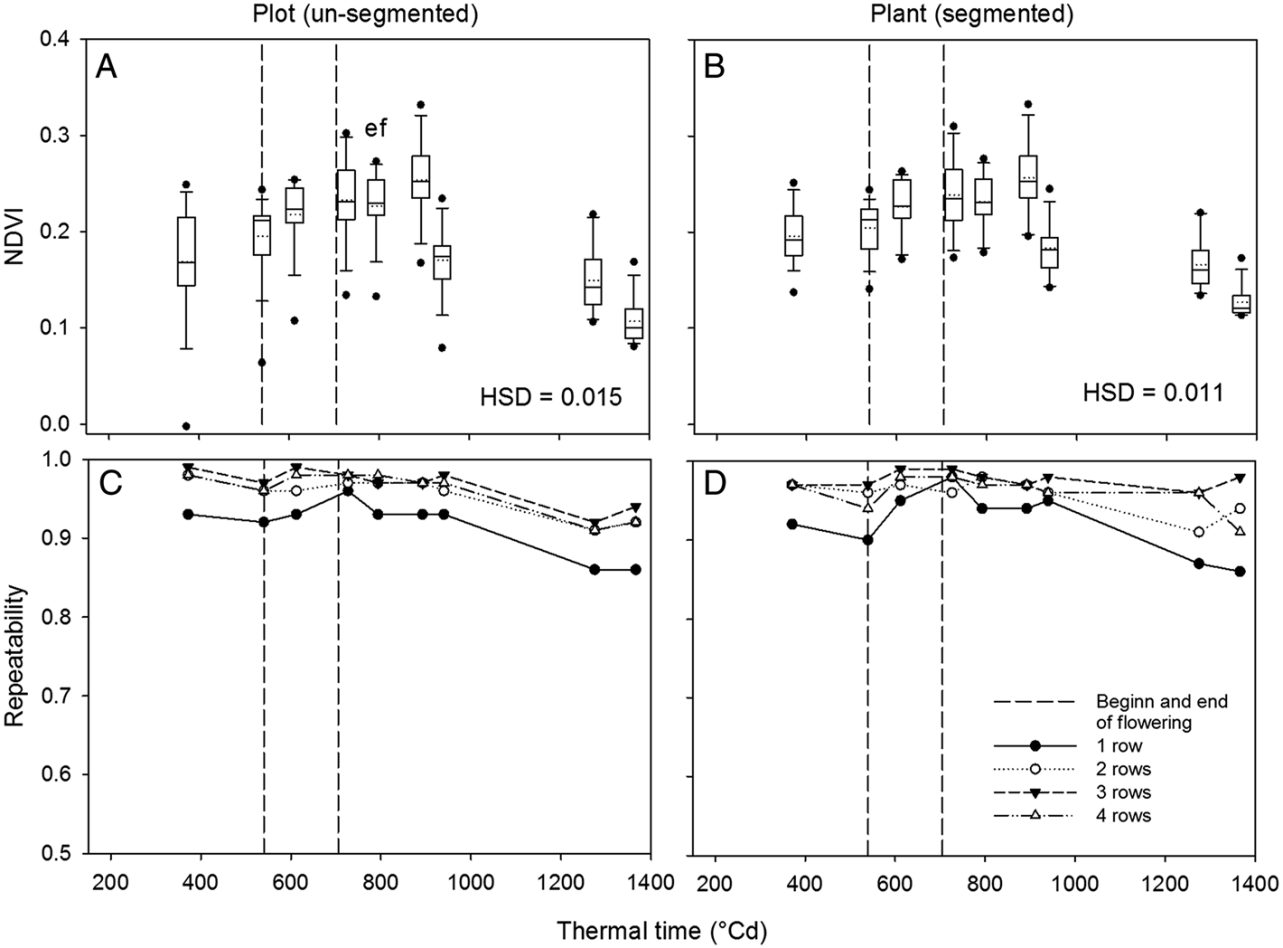
**Plot and plant NDVI and their repeatability as affected by plot size.** NDVI is shown in four row plots **(A, B)** and repeatability of NDVI in one, two, three and four row plots **(C, D)** of NDVI_Plot_ **(A, C)** and NDVI_Plant_ **(B, D)**. Boxplots are based on mean values of the 16 genotypes. The solid line in the box indicates the median and the dotted line the mean.

NDVI_Plot_ and NDVI_Plant_ (Figure 4) showed similar seasonal trends, but NDVI_Plant_ had less variance within each measurement point in time. Yet, differences were higher for NDVI_Plant_ (indicated by lower HSD), demonstrating the value of image segmentation. In general, NDVI increased until 892°Cd (Figure 4), whereas for some genotypes a plateau in NDVI values was observed from 727°Cd onwards (data not shown). At 940°Cd, NDVI dropped and subsequently decreased slowly to the lowest values observed at 1366°Cd.

The repeatability of NDVI values were high (h^2^ > 0.85) with the highest values being observed during and after flowering (Figure 4). Repeatability was generally higher in plots with more than one row, but there was only a minor advantage of having more than two rows. The reduction of the repeatability of NDVI_Plot_ towards the end of the growing season was not observed for NDVI_Plant_ in the three and four row plots.

The skewness of NDVI (Additional file 2 section 6) showed a different seasonal pattern than NDVI being relatively constant with a small reduction at the beginning of flowering and a stronger increase at the end of the season. The repeatability of the skewness was generally lower than the one for the CC and NDVI. Plot size effects were more pronounced making it less suited to differentiate among genotypes.

### Highest repeatability of canopy temperature was found on temperate days

**Figure 5:**
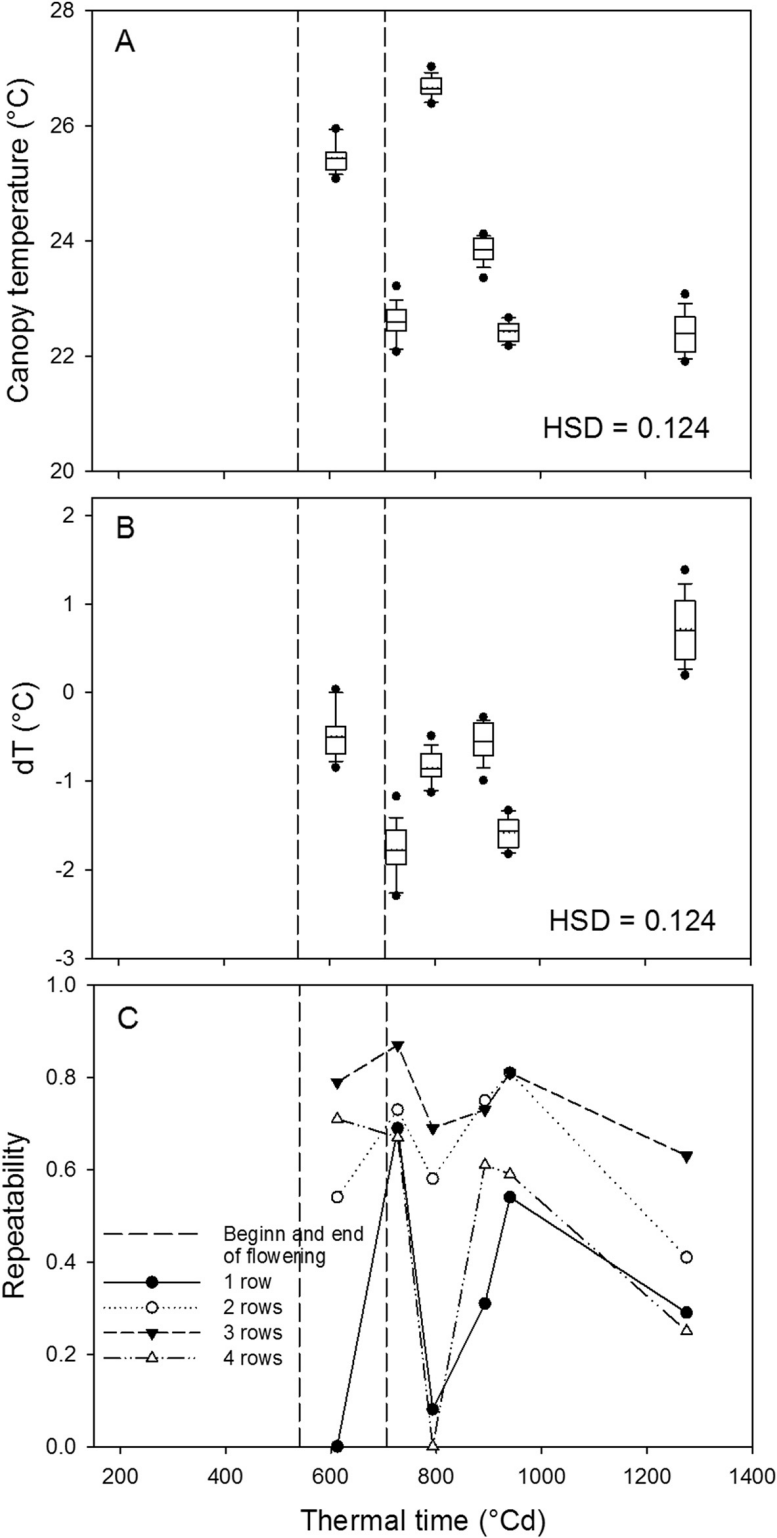
**Canopy temperature (A), difference of canopy temperature to air temperature (B) and their repeatability (C).** A + B shown for four row plots and the repeatability of one, two, three and four row plots **(C)**. Boxplots are based on mean values of the 16 genotypes. The solid line in the box indicates the median and the dotted line the mean.

Canopy temperatures (T_C_) ranged from 22 to 27°C during flight campaigns with significant differences between day of measurement (Figure 5). Across the season, T_C_ was highly correlated to air temperature T_A_, measured by the close-by weather station (Table 3). To exclude the temperature effect, T_C_ was normalized to T_A_ resulting in the temperature difference dT. As to be expected the relationship of dT to T_A_ was not significant but the effect of radiation parameters (actual PAR and sun hours) and evapotranspiration (ETo) were more pronounced (Table 3). Measurements of dT taken between 612 and 940°Cd were negative, indicating the canopy was cooler than ambient air measured by the weather station. Only at 1275°Cd when plants were in an advanced senescent stage dT was positive. The skewness of T_C_ showed a small but significant reduction over time but no genotype effect (data not shown).

**Table 3:** **Correlation coefficients determined for canopy temperature and dT with climate conditions measured at the same time, as daily averages and as cumulated values for precipitation**

**Climate parameters**		**Canopy temperature**	**dT**
		**°C**	**°C**
Canopy temperature (T_C_)	°C	1 ***	0.72
Air temperature (T_A_)	°C	0.96 **	0.51
Daily maximum temperature (T_M_)	°C	0.93 *	0.7
Gust speed	m s^−1^	−0.58	−0.14
Vapour pressure deficit (VPD)	kPa	0.23	−0.06
Actual radiation	μmol s^−1^ m^−2^	0.68	0.89 *
Precipitation (7 days cumulated)	mm	0.26	0.44
Evapotranspiration (ETo)	mm day^−1^	0.45	0.88 *
Sun hours	h day^−1^	0.74	0.97 **

Thermal data from das 1275°Cd, was excluded because plants showed already advanced senescence.

Significance codes are: ‘***’ p-value < 0.001, ‘**’ p-value < 0.01 and ‘*’p-value < 0.05.

The repeatability of T_C_ was highly affected by plot size and day of measurement (Figure 5) which differed in the prevailing climate conditions (Table 4). In one row plots, T_C_ showed a very low repeatability except at 727 and 940°Cd when high values were found in plots of all sizes (Figure 5). Interestingly, the highest repeatability values of 0.65 to 0.85 were observed in the three row plots. Surprisingly, the highest repeatability was not observed on the hot days, but on the two days with the lowest T_C_ (Additional file 1: Figure A10). At these days, we observed the strongest cooling effect of the canopy compared to ambient temperature reflected by dT.

**Table 4:** **Selected weather conditions prevailing at the time of thermal image capture (A) and some daily average values (B)**

**Date**	**year-month-day**	**2011-07-11**	**2011-07-26**	**2011-08-02**	**2011-08-12**	**2011-08-16**	**2011-09-15**
**Time**	hh:mm:ss	17:45:38	16:45:38	17:25:38	17:25:38	17:25:38	14:25:38
Days after sowing	days	81	96	103	113	117	147
Thermal time^a^	°C days	612	727	793	893	940	1275
**A at time of thermal capture**							
Air temperature (T_A_)	°C	25.95	24.40	27.52	24.40	24.01	21.71
Relative humidity (rH)	%	51.25	37.75	53.25	56.25	69.75	56.25
Dew point	°C	15.1	9.1	17.2	15.1	18.2	12.6
Wind speed	m s^−1^	0.93	1.3	0.74	1.3	0.56	0.93
Gust speed	m s^−1^	2.23	3.15	1.86	3.53	2.23	2.41
Soil temperature in 5 cm depth (T_S_)	°C	20.63	17.34	19.20	18.70	20.15	17.32
Vapour pressure deficit (VPD_air_)^b^	kPa	0.62	0.76	0.62	0.54	0.37	0.50
Photosynthetically active radiation (PAR)^c^	μmol s^−1^ m^−2^	1422	1082	nd^d^	1611	1043	nd
**B based on daily data**							
Maximum air temperature (T_MAX_)	°C	27.3	25.0	28.1	26.1	26.1	21.9
Evapotranspiration (ET_O_- Penman)^e^	mm day^−1^	4	3.1	3.7	4.5	3.1	2.1
Radiation^e^	kWh m^−2^	7.0	5.4	6.5	5.5	5.4	4.6
Sun hours^e^	h	12	9.0	11.0	11.0	10	9.0
7 day cumulated precipitation	mm	40.6	37.0	30.4	36.0	22.8	24.8
Total cumulated precipitation	mm	230.2	329.8	360.2	415.4	434.4	508.0

^a^TT = ∑if ≥ 0((T_max_ + T_min_)/2)- T_base_, T_base_ of 8°C, ^b^calculated according equation, ^c^measured with a line quantum sensor, ^d^not detected, ^e^provided by the meteorological service (LTZ, Baden-Württemberg, Germany).

### Relationship of remotely sensed parameters to ground measured plant properties

The observed maize development can be divided into three phases that can be distinguished by NDVI measurements: 1) early development until canopy closure (up to 540°Cd), 2) flowering and early senescence (540–793°Cd) and 3) late senescence up to maturity (after 793°Cd). We used different ground truth measurements depending on the developmental phase to evaluate the value of remotely sensed parameters (Table 5). For canopy cover, we considered ground truth measurements related to canopy structure and architecture but evaluated also early vigour and stay green. During the early phase, i.e. at the single measuring campaign between 303 and 371°Cd, the remotely sensed CC was highly correlated to plant density (r = 0.67) early vigour rating (r = 0.77) and leaf size (r = 0.67). During flowering and early senescence, CC was closely correlated to plant density (r = 0.73) and radiation interception (r = 0.75 to 0.86) but less to total plant biomass and leaf area index (r = 0.22 – 0.38). During senescence, CC was again closely correlated to radiation interception measured at the early senescence phase (893°Cd; r = 0.6 to 0.71) but less to late radiation interception (1275°Cd; r = 0.2 to 0.49). A correlation between stay green rating and CC was only detected during very late senescence (r = 0.36 to 0.53). No correlation was found for leaf biomass.

**Table 5:** **Selected coefficients of correlation between crop traits measured in the field and remote detected canopy cover and NDVI**_**Plant**_ **shown for different times of measurement**

**Ground plant parameter**		**Canopy cover**							
	**DAS** ^**a**^	**56**	**75**	**81**	**96**	**103**	**113**	**117**	**147**
Plant density	29	0.67***	0.78***	0.73***					
Plant vigour rating	47	0.77***	0.58***	0.48***					
Leaf length	49	0.67***	0.51***	0.34**					
Radiation interception	75	0.77***	0.74***	0.76***					
Radiation interception	97			0.82***	0.75***	0.86***			
Total plant biomass	81-97			0.34*	0.22 ns	0.3*			
LAI^b^	81-97			0.32*	0.3*	0.38**			
Radiation interception	112					0.6***	0.63***	0.71***	
Stay green rating	117					−0.22 ns	−0.2 ns	−0.16 ns	
Stay green rating	147						−0.33**	−0.12 ns	0.34**
Radiation interception	147						0.2 ns	0.49***	0.41**
		**NDVI**_**Plant**_							
Plant density	29	0.37***	0.52***	0.39***					
Plant vigour rating	47	0.64***	0.55***	0.34***					
Radiation interception	75	0.47***	0.62***	0.40***					
SPAD	96			−0.45***	−0.59***	−0.55***			
Radiation interception	97			0.49***	0.29*	0.50***			
Leaf biomass	81-97			0.39***	0.43***	0.35***			
Plant height	81-97			0.41***	0.18 ns	0.37**			
LAI	81-97			0.58***	0.44***	0.58***			
SPAD	103					−0.54***	−0.50***	−0.50***	
Radiation interception	112					0.43***	0.27***	0.38***	
Stay green rating	147						0.38**	0.36***	0.53***

^a^Days after sowing, see Table 2 for conversion in thermal time, ^b^Leaf area index (m^2^ m^−2^).

Significance codes are: ‘***’ p-value < 0.001, ‘**’ p-value < 0.01 and ‘*’p-value < 0.05.

For NDVI_Plant_ we considered ground truth data related to leaf greenness, senescence and canopy size (Table 5). During the early phase, i.e. at the single measuring campaign between 303 and 371°Cd, early vigour, was highly correlated to NDVI_Plant_ (r = 0.64). At the flowering phase, correlations to leaf biomass, plant height and leaf area index were moderate to high (0.29 - 0.58). At advanced senescence, a positive correlation to stay-green rating was observed (r = 0.53). The most striking result was the moderate negative correlation with SPAD values throughout the season (r = −0.45 to −0.59), where a strong positive relationship would be expected. This strong discrepancy may be related to camera-based constraints or to the influence of plant architecture.

For the differences in canopy temperature, negative relations were observed with radiation interception, crop vigour rating, biomass, height and LAI (Table 6). Positive correlations were found for stay green rating and leaf temperature (LTMP) during late stages of development (>940°Cd). We found no correlations with leaf stomatal conductance (LSC) and LTMP before 940°Cd most likely caused by the large time difference between ground and aerial measurements.

**Table 6:** **Selected coefficients of correlation between crop traits measured in the field and remote detected temperature difference (dT) shown for different times of measurement**

**Ground plant trait**		**dT**				
	**DAS** ^**a**^	**81**	**96**	**103**	**113**	**117**
Plant vigour rating	47	−0.45***	−0.4**			
LSC^b^	81	0.09 ns	−0.08 ns			
LTMP^c^	81	−0.16 ns	−0.1 ns			
Radiation interception	81	−0.59***	−0.55***			
Radiation interception	97	−0.55***	−0.24 ns			
Total plant biomass	81-97	−0.38*	−0.34*			
Plant height	81-97	−0.46***	−0.53***			
LAI^d^	81-97	−0.52***	−0.49***			
LSC	103			−0.07 ns	−0.27*	0.17 ns
LTMP	103			−0.06 ns	−0.19 ns	0.09 ns
Stay green rating	105			0.12 ns	0.2 ns	0.34**
Stay green rating	112			0.1 ns	0.12 ns	0.36**
LSC	117			−0.17 ns	−0.21 ns	−0.13 ns
LTMP	117			0.45***	0.53***	0.28*
Radiation interception	118			−0.52***	−0.29*	−0.66***

^a^Days after sowing, see Table 2 for conversion in thermal time, ^b^Leaf stomatal conductance (mmol m^−2^ s^−1^), ^c^Leaf temperature (°C), ^d^Leaf area index (m^2^ m^−2^).

Significance codes are: ‘***’ p-value < 0.001, ‘**’ p-value < 0.01 and ‘*’p-value < 0.05.

### Genotypic differences

**Figure 6:**
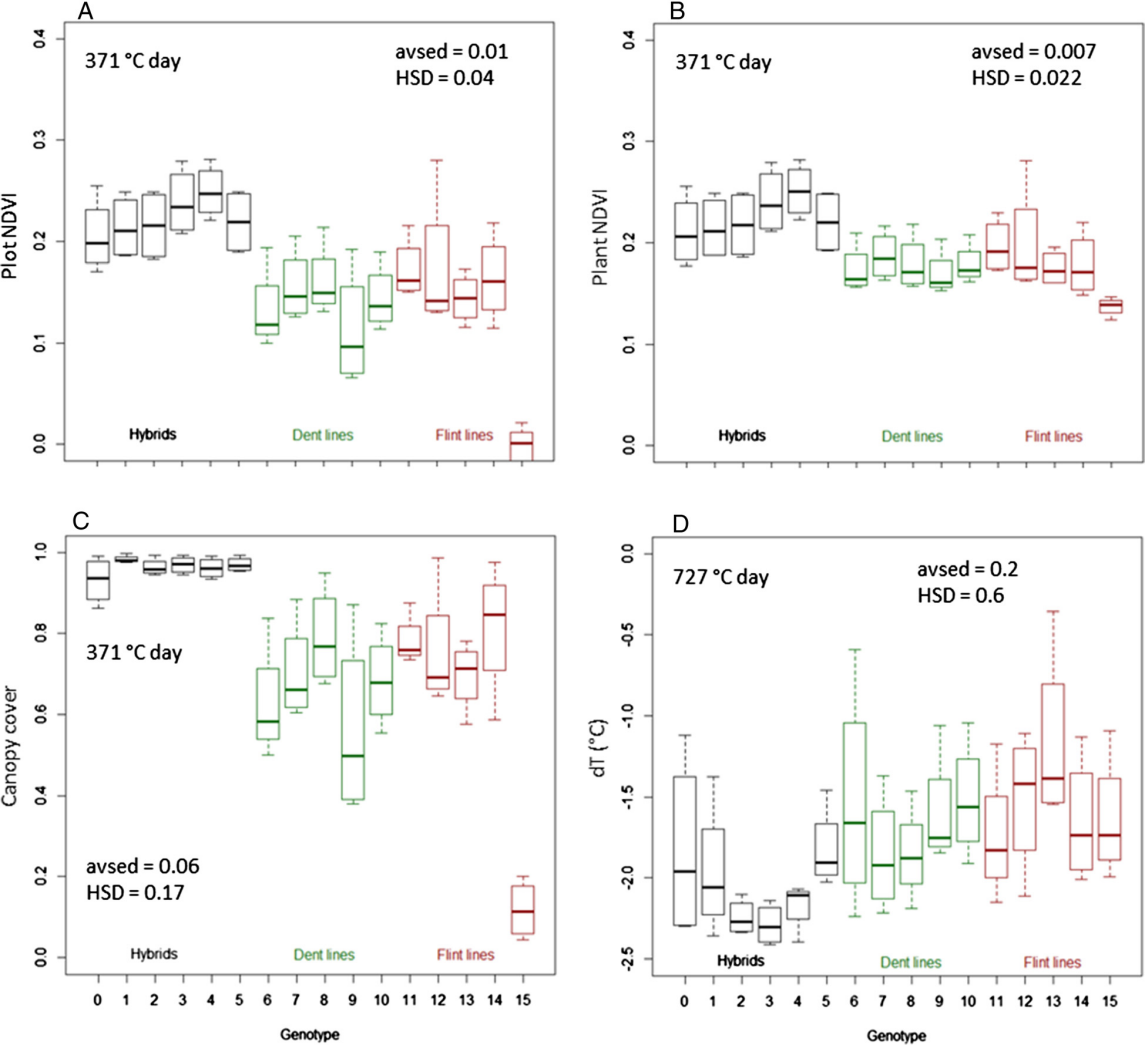
**Genotypic differences documented by aerial imaging.** NDVI_Plot_ **(A)**, NDVI_Plant_ **(B)**, canopy cover **(C)** in a vegetative growth stage (371°Cd) and difference of canopy temperature to air temperature (dT) **(D)** during flowering 727°Cd. HSD = honest significant difference (α = 0.05), avsed = average standard error of the difference. Colour of boxplots indicates maize type: hybrids (black) or maize ideotype of inbredlines: dent (green) and flint (red). The shown dates were based on observed significant differences between genotypes and relevance of the particular growth stage for plant breeding purposes.

Clear differences between genotypes were detected for all parameters (both in remotely sensed parameters and ground-truth traits). A detailed discussion of these effects would exceed the scope of this manuscript and will be done in a future publication. Yet, in order to demonstrate the value of the presented method, it is important to point out a few cardinal differences detected for the remotely sensed parameters (Figure 6). For CC, differences between hybrids and inbred lines were pronounced because of the larger canopies of the hybrids. Their canopies were mostly closed in a 6 to 9 leaf stage (at 371°Cd), when the CC of the inbred lines was still below 0.9 (Figure 6). At the beginning of tasseling (540°Cd; data not shown), the majority of the genotypes had a CC higher than 0.9. Evaluating CC of the inbred lines, we observed genotype 9 to be significantly lower than the genotypes 8, 11, 12 and 14.

the genotypes differed for both, plant size and CC, NDVI_Plot_ clearly distinguished among genotypes. However, NDVI_Plant_ showed the difference of leaf greenness independent of differences in CC due to emergence rates or canopy architecture. This effect was clearly found for the three genotypes 6, 9 and 15 with plant densities below 70% of the original sowing rate (data not shown), where NDVI values markedly increased when measured on a plant basis instead of a plot basis. It is clearly visible that hybrids had generally higher NDVI_Plant_ values compared to inbred lines.

Significant differences of T_C_ between genotypes were found at 612, 727, 893 and 940°Cd, but not on 793 and 1275°Cd (data not shown). On 793°Cd we measured the highest T_A_ of 27.5°C during IR image acquisition. However, it did not lead to a good separation among genotypes in contrast to dT. At flowering (727°Cd) when T_A_ was lower, T_C_ ranged between 22.0 and 24.0°C and were 1.4 to 2.3°C lower than T_A_ depending on genotype (Figure 6). The highest dT was found for genotype 3 and the lowest for genotype 13.

## Discussion

### Imaging platform, sensors and experimental field site

Many non-destructive measuring techniques are ground based or stationary using fixed, handheld or motorized systems (e.g. tractor mounted sensor platforms or crane systems). Thus, they are often limited to relatively small measurement areas and low numbers of replicates or genotypes. Furthermore, they are rather labour and time intensive, and do seldom cover temporal plant development [1,14,15]. For example, three tractors and several workers would be needed to measure a typical breeding set of 20’000 plots in a few hours [1]. In contrast, aerial remote sensing offers the potential to cover large areas planted with many plots in relatively short time. In our study, the experimental field of 0.4 ha (30 × 132 m) was imaged from the air within 10 s. Accordingly, it would take around 6 minutes to monitor 20’000 two row plots of 1.5 × 4.75 m covering an area of 14.25 ha. Of course, additional time might be required depending on allocation, alignment and shape of the field.

The Zeppelin proved to be a valuable remote sensing platform due to the limited restrictions for sensor weight and its slow speed during image acquisition. Too high travel speeds can cause blurring effects and thus mix target and non-target information deteriorating the quality of the measurement. With a cruising altitude of 300 m at the highest speed of 20 km h^−1^, the lowest image resolution (thermal camera) was 10 × 10 cm of ground cover per image pixel. During the opening of the shutter of 50 ms, the thermal camera moved 0.28 m along the row resulting in a blurring effect of 3 pixels. In order to keep the blurring effect within the row, it is preferable to fly in row direction rather than crossway of it. That blurring effect was less affecting the cameras with higher resolution and shutter speed and is reduced by lower cruising speed.

Costs of the Zeppelin operation were low compared to tractor-based operation, since a touristic route was used and only one man-hour plus ticket costs were required to take the images. Of course, this makes the choice of the test location extremely inflexible. Limits of the Zeppelin system are mainly the weather conditions and the mission area. Weather conditions restricting flights are mainly wind, rain and thunderstorms. Wind speeds above 25 m s^−1^, and thunderstorms keep the Zeppelin on the ground or force the pilots to return to the air field (personal communication with Zeppelin NT). Rainy conditions do not interfere with the ability and allowance to operate the Zeppelin but affect data values of the image due to high water content in the light path [16]. In the beginning of the experiment the unpredicted occurrence of bad weather conditions prevented image acquisition during the early growth stages of maize.

The shown remote phenotyping approach may be adapted to other aerial platforms such as blimps, fixed wing or helicopter drones or even planes. A review of platforms and sensors would exceed the focus of this paper (for an overview of platforms see [16] for sensors [17]). However, drones combined with lower weight sensors and sensing technology as used in precision agriculture [18] seem to offer the temporal and spatial flexibility needed for phenotyping (for a review on drones see [19]). Because of pay load restrictions most drone approaches use a single sensor or sensors restricted in measurement capabilities. Remote sensing platforms capable of carrying a high payload and thus multiple or high weight sensors such as large drones and air planes are restricted in use by issues such as costs, law, region, manpower and training [19] similar to the Zeppelin platform. Additionally, the high flying altitude and speed resulting in high ground pixel sizes limits their use for crop phenotyping. In the future, light weight or micro drones combined with low weight sensor technology as currently investigated for precision farming applications might enable flexible high throughput crop phenotyping with multiple sensors and high temporal resolution as needed for breeding research.

The field markers that were used to semi-automatically match the images from the different sensors were an important feature of the experiment. Their identification was the prerequisite for the semi-automated registration of the area of interest and the ortho-correction process. Detection of the markers in RGB and NIR images may be improved by including white centres on the black plates, but it is not yet clear how that would interfere with thermal detection. Single plot labelling, as described by Jones et al. 2009 [20] for ground IR imaging would require too much investment for large, remotely sensed field setups. We consider markers to identify the corners and intermediate way points of the experimental area as sufficient to correct for image distortion and to allow for a correct positioning of a plot map.

Once the processing pipeline was established, data processing needed relatively little labour for input file conversion, check of the correct identification of the field markers, identifying and setting thresholds for segmentation and for creating the plot overlay. Time needed for conversion of proprietary input files (*cr2* from Canon and *irb* from InfraTec) into the open tagged image file format (*tiff*) can be minimized by using acquisition software saving in open formats in future measurement campaigns. Nevertheless, a manual inspection of the correct identification of the field markers was necessary. It was facilitated by an automatically created overview and by a manual interface to identify the centre of the field marker, if necessary. Further development of reliable field markers in combination with suitable software will be necessary to improve automation for large areas monitored with higher throughput.

### Image segmentation to distinguish into canopy cover and the NDVI value of the canopy itself was useful

The threshold settings for image segmentation for the seasonal imaging campaigns were similar for the NDVI but had to be adjusted for grey intensity (shades vs. non-shade) according to radiation condition prevailing during image capture. This procedure may be optimized by using the relationship of global radiation to grey intensity threshold in future campaigns. However, changing canopy properties might complicate that. Another option could be the placement of additional reference markers with different grey scales or colour to enable subsequent adjustment of the exposure values. This would also enhance the comparability of values between different flight campaigns.

Segmentation in images above mm scale resolution does always result in mixed pixels of either plant or soil features along edges or feature borders. A rule of thumb is, that for precise object identification a minimum object size of three times the instantaneous field of view (IFoV), i.e. 3 ground pixels is needed [21]. Considering an IFoV of 2.5-3 cm for the vegetation camera, it is evident that three pixels were not available for object identification in regions of leaf tips, edges and where tassels cover the leaves. Such impurities were apparently marginal influential for determination of CC in both RGB and B-NIR images but should be minimized to a certain degree e.g. by using cameras with higher resolution. In our case the detection of CC appeared relatively robust, particularly CC was higher than 0.5. However, we were not able to detect young seedlings, which had approximately two fully developed leaves (first flight data not shown). Accordingly, an improvement is to be expected by using higher resolution cameras, especially to estimate germination rate, development during early growth stages, or if the detection of changes in tassel colour is anticipated in order to determine the time of flowering.

We used image segmentation to generate two independent parameters: CC to measure the canopy cover and NDVI_Plant_ to measure leaf greenness independent of differences in canopy cover. The alternative, average NDVI signal of the whole plot without segmentation reflects a combination of temporal, spatial and genotypic variation of leaf greenness and CC. Thus, NDVI_Plot_ should be interpreted carefully if used for plant phenotyping. Only when canopies are closed differences in leaf greenness measured on plot base can be considered reliable. Here the skewness of NDVI_Plot_ can be used as a quality parameter with values higher than zero indicating plots with lower CC. At lower CC, NDVI_Plot_ is to a large extent influenced by soil pixel and, thus, information about leaf greenness is masked by differences in CC (see Additional file 2, section 6 for details). Thus, detection of leaf greenness reduction during senescence based on NDVI_Plot_ must be hampered as soon as CC becomes sparse.

We also tested, whether the distribution parameter of the skewness of the NDVI_Plant_ would be valuable as indicator for senescence. Senescence increases the patchiness of green, yellow and brown leaf parts [22]. In our study, the explanatory power of skewness was limited because effective pixel size was too small to disentangle soil and plant signal sufficiently (see Additional file 2 section 6).

For the detection of NDVI_Plant_, even one row plots were sufficient but clearly two and more rows improved repeatability further. We consider two row plots as a good balance between the precision to measure genotypes remotely and the necessity to screen large numbers of genotypes.

### Thermal imaging had too little resolution for a suitable segmentation

For the thermal images no effective segmentation could be conducted because of the large IFoV of 0.3 × 0.1 m (discussed above). It was possible to detect maize rows, interspaces and larger patches of soils but no single leaves (Additional file 2). Therefore, the investigated whole plot signal reflects a plant and soil mixture. Accordingly, it is important to take canopy cover into consideration when comparing among genotypes. Similar observations were reported by Jones et al. [20] and Costa et al. [23]. Thermal measurements with the here reported ground resolution are applicable without restrictions in crops with closed canopies or in orchards, where plant area and unplanted inter row spaces are large and generally have a different temperature than the targeted plants as in [15]. Using the Zeppelin, an increased resolution can be achieved by reduced flight altitude (down to 80 m) and speed (down to 0 m s^−1^). This would bring the ground resolution below 0.03 by 0.03 m instead of 0.1 by 0.3 m as in the present study. Alternatively, software solutions such as multiframe super resolution ([24]) or sensors with a higher resolution are an option.

Due to the mixed signal, the repeatability of T_C_ was higher in larger plots. The partially observed low repeatability of T_C_ in the four row plots is assumed to be caused by the intensive use of the four row plots for regular ground truth measurements. These frequent activities led to broken lower leaves and may have compacted the soil in the inter-row spaces leading to additional random noise. This indicates that entering plots for thermal measurement on a regular base should be avoided.

### Seasonal development of remotely sensed traits

The observed development of CC and NDVI_Plant_ as an indicator for leaf greenness in this study is in agreement with many studies, which recognized two to three phases of maize development depending on sensor type and parameter used [25-27]. In this study the initial growth phase was only represented by one flight campaign but the early vigour rating was still well related to the measurements at the end of this phase. This indicates the possibility to phenotype early growth stages of maize by remote sensing. Thereby the limit of early growth measurements is defined by the IFoV as discussed above and by plant size. In this study, plants were sufficiently large from the 4 to 6 leaf stage onwards. The following phase with relatively constant and high NDVI corresponds to a so-called plateau phase [26]. The duration of this phase and the height of the NDVI values depended on the genotype-specific flowering time and beginning of senescence. The last phase of maize development was well identified by a decrease in NDVI_Plant_ and its skewness following progressive senescence. The late start of the senescence detected by remote sensing as compared to rating on the ground, can be related to the fact that senescence starts at lower, older leaves [25] which are not detectable from above. Nevertheless, the NDVI parameters did reflect stay green rating as the inverse of the senescence after 900°Cd.

### Correlation of image-based parameters and plant traits

We aimed to evaluate whether the remotely sensed parameters, NDVI, CC and T_C_, reflect plant traits measured on the ground, like biomass, radiation interception, plant density and plant vigour.

Measurements of CC during early maize development and after beginning of corn filling, where genotypic variation was highest, seem to be promising indicators for early vigour and delayed senescence. Although very early measurements are missing in this experiment the derivation of CC from aerial images was successful and relationships to ground truth parameters were validated. The strongest correlation was found to radiation interception which itself is a strong non-destructive indicator for canopy size and leaf area traits. Clearly, the measurement of canopy coverage and density in the field is very time consuming compared to the aerial approach [28], justifying its application for large populations.

NDVI_Plant_ appeared to measure the opposite of leaf greenness, as measured by the SPAD meter. This negative relationship was not expected because SPAD values indicate leaf greenness as a function of chlorophyll content (Additional file 2: Figure A4) and thus should be positively correlated to NDVI values [11,29]. Such a negative relationship of camera-based vegetation indices to SPAD was also observed by [30] who reported them to be in contrast to narrow or broad band indices. The camera channels B and NIR of the vegetation camera used in this study covered a range of 370 to 480 nm for the blue channel and 675 to 775 nm for the near infrared channel, respectively. The SPAD values are measured as transmission difference of two narrow bands (<10 nm range), with a high chlorophyll absorbance at 650 nm and a low absorbance of chlorophyll at 940 nm with a stable light emission by a red and a NIR LED (Konica Minolta Sensing Inc., Osaka, Japan). Accordingly, the NDVI camera uses a much broader range of the spectrum and different wavelengths than the SPAD measurement. Measurements with more precise narrow-band imaging sensors will likely improve the detection of leaf greenness as related to leaf chlorophyll content with spectral indicators such as NDVI_Plant_.

Despite the failure to measure leaf greenness as a function of chlorophyll content as observable with the SPAD meter, we believe that the strong correlations of NDVI_PLant_ with other plant characteristic support the applicability of this method for breeding approaches. Its correlation to the leaf area index around flowering is very useful for remote-sensing applications, especially as canopy cover was little related to the leaf area index during this phase. Plant density and early vigour reflect germination rate and the genotypes’ ability for fast establishment in the field, respectively. These two important traits which describe early development of maize are used for breeding purposes [31]. Stay green is a trait reflecting the plant’s ability to maintain the photosynthesis functioning in the final growth stage. It is linked to increased yields as well as enhanced stress tolerance [11,32,33].

The effect of transpiration cooling of plants can be shown by the normalization to standard temperatures such as T_A_ or temperatures of certain standard surfaces [20]. We measured T_A_ with a weather station resulting in reasonable dT values: when maize was imaged in a green and transpiring stage, the cooling effect was −0.5 to −2°C; when it was imaged in the senescent non-transpiring stage, dT was lightly positive. Leaf temperatures can also be higher than T_A_ when radiation intensity is very high (e.g. at noon) and wind conditions are stagnant as shown in lab and field studies [9,34]. In this study, high radiation conditions during remote measurement campaigns were avoided due to late afternoon flights when radiation is lower. At the conditions presented here, the temperature normalization (dT) enabled a meaningful comparison between genotypes, measurements on different days with different climate conditions.

Highest repeatability, i.e. best differentiation among genotypes was achieved at days with moderate T_A_ when the largest cooling effect of the canopy was observed. This observation is in contrast to studies were T_C_ is measured mostly during hot days at the hottest time of the day around noon as an indicator for drought tolerance adaptation of genotypes [8,9] or crop water status [35]. Most of such studies were conducted in a different climate and with different research questions than this study and thus cannot fully be compared. Certainly, the optimal time of the day and temperature for IR measurements for plant breeding might still be a question to be answered. Due to unfavourable thermic conditions in the target area at midday, the company operating the Zeppelin did not allocate regular flights to the area where the experiment was placed. This made it impossible to test which time of the day would be optimal for thermal imaging.

The strong, negative correlation of dT with plant size and coverage information such as radiation interception, LAI and biomass confirms the applicability of the IR camera to measure T_C_. In canopies with higher biomass, coverage and plant density T_C_ is lower reflecting a higher transpiring area and cooling effect. Additionally, the correlation to radiation interception and LAI may be explained by the large pixel size and thus the mix of soil and plant information in the signal. The low correlation of T_C_ and dT to stomatal conductance and leaf temperature measured with the porometer might be explained by methodical differences as well as genotypic differences. The porometer measurements in the field reflect two point measurements per plot at the youngest fully developed leaf in a four row plot and thus only a marginal part of the IR plot image. This is supported by the observation of a better correlation of T_C_ to leaf temperature in the later growth stages when stomatal conductance is reduced due to advancing senescence and, thus, genotypic properties affecting T_C_ are less important. The positive correlation of Tc to the stay green rating may be an effect of differences in CC and canopy architecture properties. A lower CC results in lower leaf area as well as higher soil area in the image and thus a smaller dT of the canopy.

## Conclusion

We developed a multi-channel remote-sensing pipeline with semi-automated image analysis.

The comparably low cruising altitude and cruising speed of the Zeppelin combined with high ground resolution enabled image segmentation. Accordingly we could distinguish into canopy cover (CC) and the normalized difference vegetation index of the segmented canopy (NDVI_Plant_). Such segmentation was not possible for the thermal images with their comparably lower resolution. For CC and NDVI_Plant_, two row plots enabled a sufficient differentiation among genotypes; for thermal imaging, more than two rows are preferable.

The NDVI camera could be used to measure different traits, depending on the time of the year. Early in the season, CC was related to early vigour, leaf length and plant density, later it was related to radiation interception. NDVI_Plant_, was well related to the vigour rating and to very late senescence rating. More important, it was related to the leaf area index during flowering, when canopy cover did not correlate well with the trait. Most strikingly, NDVI_Plant_ was negatively related to leaf chlorophyll content measured with the SPAD-meter. This discrepancy demands for an in-depth evaluation of this phenomenon.

For the thermography, highest repeatability of canopy temperature was observed on large plots on temperate days with strongest differences in canopy cooling.

The presented aerial phenotyping approach is applicable to other crops and larger field experiments and genotypic sets as well as other aerial carrier and sensor systems. Similar approaches might be realistic with light weight aerial carriers in the future when sensor technology evolves and sensor weight decreases, especially for thermal imaging. Such approaches can help to close the gap between phenotyping and genotyping and reduce the constraints currently limiting breeding advances.

## Method

### Experimental set up

The experimental field was placed below one of the frequently operated touristic routes of the Zeppelin (Zeppelin NT, Friedrichshafen, Germany) in the area of Lake Constance. It was embedded in a maize field near Salem in Germany (47° 46’ 15.37” N, 9° 17’ 15.16” E, 440 m.a.s.l.). The soil was a cambisol [36] classified according to soil texture as a sandy loam. The experimental setup was organised as a split plot design with four replications, the number of rows per plot (one to four) as the whole plot factor and a set of 16 genotypes as the split plot factor (Additional files 1 and 2). To avoid neighbour effects between hybrids (entries 0 to 5) and inbred lines (entries 6 to 15), the two groups were randomized in two separate blocks within the split plots. The plot length was 4 m and row spacing was 0.75 m. Additional single row plots of 10 m length were created at the end of each block for destructive samplings and measurements.

For the precise detection of the experimental plots in the aerial imagery we placed field markers within and around the experimental field (Figure 1). Two types of markers were used. Nine white plastic tarps (1 × 2 m) were placed on the ground in diagonal cross-like form just after sowing. Eight round, black metal plates (Ø 70 cm) were placed on top of 2 m poles along the edge of the experimental field after canopy closure.

### Maize genotypes and cultivation

We selected 16 maize genotypes which reflect a large variability in plant development and morphology. The selection comprised six commercial hybrids (entries 0 to 5), five dent (entries 6 to 10) and five flint inbred lines (entries 11 to 15). The genotypes were Lapriora (entry 0, KWS SAAT AG, Einbeck, Germany), DKC2960 (entry 1, DeKalb Genetics Corp., Dekalb, IL, USA), Tiago, Pralinia, Bonfire, Swiss301, DSP1771, DSP5009S3, DSP5049A31, DSP5145X1, DSP5164A3, DSP2563E3, DSP2637A (entries 2 to 12, Delley seeds and plants, Delley, Switzerland), UH003 and UH008 (entries 13 and 14, University of Hohenheim, Germany) and SMxxx (entry 15, Freiherr von Moreau Saatzucht GmbH, Altburg, Germany).

The genotypes were planted on April 21, 2011 with a planting density of 9 plants per m^2^ using a single-seed drilling machine (type TRM, Wintersteiger AG, Austria). The maize was cultivated according to best management practices by the local farmer (for details see Additional file 2). For spraying of pesticides a tractor mounted wing sprayer with a wing length of 15 m was used (no crossing through the experiment).

### Climate and weather conditions

Air temperature, relative humidity (2 m above ground), precipitation, wind- and gust speed (3 m above ground) and soil temperature (5 cm in the soil) were recorded with an on-site weather station (Onset Hobo, Pocasset, USA) installed at the edge of the field, at a distance of 150 m to the experiment. Thermal time (TT) was calculated as TT = ∑_if≥0_((T_max_ + T_min_)/2)- T_base_, with a base temperature (T_base_) of 8°C [37] and is expressed in degree days (°Cd). Vapour pressure deficit (VPD) was calculated as VPD = ((100 - rH)/100)*SVP, with the saturation vapour pressure: SVP (Pa) = 610.7*10^7.5T/(237.3+T)^.

Additionally, for days with thermal measurements evapotranspiration (ETO- Penman), radiation and number of sun hours were derived from a close by commercial weather station at Ailingen (47° 41’ 30.49” N, 9° 28’ 11.79” E, 440 m.a.s.l.) managed by the local meteorological service (LTZ, Baden-Württemberg, Germany).

### Aerial imaging equipment

In this experiment we used a Zeppelin operated by Zeppelin NT (Deutsche Zeppelin-Reederei GmbH, Friedrichshafen, Germany) as remote sensing platform. In this proof of concept study, we decided to buy tourist tickets and to acquire images out of the open side window instead of a fixed on-board installation of our equipment. A fix installation would have demanded for an aviation certification and training for the pilots for using the imaging equipment. The sensor array was secured against falling off. During flight campaigns (Table 2) the Zeppelin was directed along the experimental field in south to north direction. Images were captured at approximate nadir position (view angle 90° to the soil surface) at an altitude of about 300 m and cruising speed between 0 and 20 km h^−1^ depending on wind situation.

For image capture we used a handheld camera system (Figure 1), which consisted of two consumer grade cameras and an optionally attached thermal camera. The consumer grade cameras were a 10.1 megapixel CMOS RGB camera (Canon EOS 400D, Canon, Tokyo, Japan) and a two-channel, 12.2 megapixel CMOS vegetation camera (Canon EOS 450D NDVI, modified by LDP LLD, Carlsted, USA) with a sensitivity range of 370 to 480 nm (blue channel, B) and 675 to 775 nm (near infrared channel, NIR). More information on sensor sensitivity and NIR photography can be found in Nijland [38]. The two cameras were equipped with Canon EF-S 60 mm f/2.8 Macro USM lenses and mounted on an aluminium frame with handles and interconnected with a remote trigger cable for simultaneous image capture. The aperture size was adjusted shortly before the field capture using a random maize field between the airport and the experimental field. The focus was centre weighed and set to AI servo mode, the ISO was set to 100 and all other settings were set to automatic. During the flight over the experimental field a series of images was taken.

The thermal camera was an industrial grade thermal infrared (IR) camera VarioCAM head 600 (Infratec GmbH, Dresden, Germany). It was attached only for selected missions on hot summer days (Table 2). The IR camera measures in the spectral range between 7.5 and 14 μm, a spatial resolution of 640 × 480 pixels and a thermal resolution of better than 0.03 K at 30°C. A 75 mm lens was attached and shutter speed was 50 ms. For mobile image acquisition the IR camera needed an additional battery and a laptop connected by fire-wire for camera control and data saving. The camera was attached to the handle bar between the two consumer grade cameras and was run in video mode recording five images per second during flight over the experimental field with the focus adjusted automatically (every 40 seconds) shortly before the field was reached.

Images were recorded in raw format (.cr2 for the Canon cameras and .irb for the IR camera). The total imaging setup had a weight of 7.2 kg, a detailed description (incl. information about the ground cover and spatial resolution for the three cameras at altitudes of 300 ± 10 m) can be found in Table 1.

### Image processing and analysis

For analysis of the aerial imagery the macro array of field plots arranged in the experimental field was split into three sub arrays (rectangles), each one covered by a separate image scene (a detailed sketch of the field plot macro array can be found in the Additional file 1). For each camera images were selected manually and transformed from the respective raw file format to 16 bit .tiff images (Figure 1C). Selection criteria were that the target rectangle was well focused and central in the image to minimize vignette effects.

The image processing scripts were developed in Matlab (2011a Natick, MA, USA). The black field markers (Figure 1D) were used to automatically identify, match and co-register the sub arrays in the different images. For the RGB and the NDVI camera the blue channel was used to identify the markers in the images. In order to accurately transform these images into the same coordinate system, the marker positions were determined consistently by normalized cross correlation (NCC) [39]. Here, the marker regions of the NIR images served as templates and their best position (most similar position) in the blue channel of the RGB image was determined in the region of the corresponding markers by NCC. In the thermal images, the black field markers emitted higher temperatures than plants and soil. However, the success of the automatic marker detection procedure was manually adjusted in a few cases. Subsequently, the marker positions were used to rectify the sub array images by applying a projective transformation using bi-cubic interpolation. For IR images the resolution was up-scaled to the resolution of the other sensors before applying the projective transformation. The result is a set of images from all sensors transformed to the same coordinates for each sub array (see Figure 1D).

For evaluation of plant features we differentiated soil from plant pixels, by means of segmentation [21]. This procedure was tested for the blue-near infrared images (B-NIR) and the RGB images separately. For B-NIR we calculated the normalized difference vegetation index (NDVI) based on the blue band instead of the red band: NDVI = (NIR − B)/(NIR + B), where B is the blue channel and NIR is the near infrared channel. For the B-NIR images the segmentation of plants was performed using two separate threshold procedures. The first segmentation was based on NDVI with a threshold of 0.1 meaning, all pixels with a higher values were regarded as plant pixels. The threshold 0.1 was chosen for all images to allow detection of maize leaves with reduced greenness particularly during the late development stages (senescence). The second segmentation step was done after converting the images to monochrome images, which shows the reflection intensity, in order to remove highly shaded areas. It was directly affected by the actual radiation and thus was set individually for each flight campaign depending on radiation conditions (thresholds can be found in Additional file 2). The resulting masks were combined by multiplication. For the segmentation of the RGB images the images were transformed to the HSB colour space. Thresholds were set for hue, saturation and brightness, respectively and for each flight individually.

To identify the sampling plots and to exclude unwanted areas such as tracks around the sampling plots, a mask file was prepared (Figure 1). The mask was used as overlay to clip and save an area of interest (AoI) for each experimental plot. For each camera and field rectangle an image stack was generated for visual control of the output images.

The per plot extracted data comprised the median and skewness (distribution parameter) for the RGB channels, the NDVI and canopy temperature (T_C_) with and without segmentation, respectively. The skewness of a distribution is a rating of the asymmetry of its histogram relatively to its distribution mean (Additional file 2: Figure A5) and is defined as:

$$ s = \frac{\frac{1\ }{n}\ {\displaystyle {\sum}_{i=1}^n}{\left({x}_i-\overline{x}\right)}^3}{{\left(\sqrt{\frac{1\ }{n}\ {\displaystyle {\sum}_{i=1}^n}{\left({x}_i-\overline{x}\right)}^2}\right)}^3} $$

where $n$ is the number of distribution elements, *x*_*i*_ is the i-th element and $\overline{x}$ is the mean. Negative values are encountered if the median of the distribution is greater than the distribution mean and positive values if it is smaller. If the distribution is symmetric to its mean, the skewness is zero.

The canopy cover (CC) was extracted as the fraction of plant pixels from the segmented NDVI images. From T_C_ we calculated the difference to air temperature (dT) using the actual air temperature (T_A_) measured by the weather station on-site at the time of the image capture.

### Maize development and ground truth measurements

Unless reported otherwise, all observations presented here, were taken from the four-row plots. Evaluations and measurements started 0.5 m behind the first plant to minimize edge effects. Emergence was evaluated on 4 m rows 155°Cd corresponding to 29 days after sowing (DAS). The corresponding dates, degree days, DAS and approximate growth stages can be found in Table 2. Tasseling was evaluated during the period from 540 to 727°Cd on ten adjacent plants in approximately 3-day intervals. The exact dates at which 50% of the plants were tasseling were determined by linear interpolation. Leaf and total above ground biomass, plant height and number of leaves were determined on five adjacent plants in the sampling plots when the respective genotype was considered fully tasseling. Fresh weight biomass was determined with an electric field balance and height was measured with a yard stick. Stay green (development or delay of senescence) was evaluated five times from 815 to 1275°Cd on ten plants per plot by counting green leaves below the ear [40].

The leaf area index (LAI), was calculated from leaf biomass taking advantage of the narrow relationship between leaf area and specific leaf fresh weight (SLW) determined on a subset of plants (n = 24, r^2^ = 0.98): LAI (cm^2^ cm^−2^) = SLW (mg cm^−2^) -3.96/27.4, with SLW cm^−2^) = leaf biomass (g)/(SL (cm)*70 cm)*1000, where SL is the sampling length and the row distance is 70 cm. Details on the sub-experiment can be found in the Additional file 2 in section 5.

Leaf chlorophyll content, canopy radiation interception and stomatal conductance were determined throughout the season mostly parallel to the aerial imaging campaign. Leaf chlorophyll content was determined with a SPAD meter (Konica Minolta Sensing Inc., Osaka, Japan) on 10 leaves per plot. Before silking, measurements were done on the youngest fully developed leaf. After silking, SPAD was measured on the second leaf above the ear leaf. Photosynthetic active radiation (PAR) was measured with a 1 m line quantum sensor (LI-186-line, LI-COR, Lincoln, Nebraska, USA). Measurements were taken in the middle row on the ground (PAR transmitted) and above the canopy (incident PAR) at noon on clear days or days with stable cloud cover. The proportion of PAR radiation absorbed by the crop (radiation interception) was calculated as the ratio of the difference between incident and transmitted PAR to incident PAR [41]. Leaf stomatal conductance (LSC) and leaf temperature (LTMP) were measured with a steady state diffusion leaf porometer (SC-1, Decagon Devices, Pullman, WA, USA) on the same leaf as SPAD. Measurements were taken in early afternoon (12–14:00) with two measurements per genotype and block for time reasons.

### Statistical analysis

The investigated dataset consisted of three levels of data: (1) genotype level: ground truth data collected in the destructive sampling plots, which were not the same as aerial survey plots, (2) plot level: ground truth measured in plots at the same day and on the same plots as the aerial survey (generally four row plots) and (3) the remote sensing level: data available for all plots and plot sizes measured at the same time. Data from different levels of measurement were combined by time of measurement (TT), genotype and block. Data consisting of more than one measurement per plot (SPAD, LSC, silking, stay-green rating, and biomass) were averaged before entering data analysis.

Statistics were calculated with R version 3.0.1 [42]. Boxplots show the 25 and 75% quantiles as the lower and upper limit of the box with the median as solid line in between (mean values shown as dotted line in some cases). The lower and upper whiskers represent the 5 and 95% percentile or the minimum and maximum value if no individual points (outliers) are plotted.

Comparisons between genotypes or measurement dates were done by means of a mixed model analysis using the package ‘asreml’ version 3.0 for R [43] followed by a HSD test. The variance components to estimate repeatability of the one to four rowed plots were determined by setting block as fixed and genotypes as random factor. Repeatability was calculated as h^2^ = σ^2^_gen_/(σ^2^_gen_ + σ^2^_ε_/4), where σ^2^_gen_ is the estimated genetic variance and σ^2^_ε_ is the residual error variance. We used the repeatability to elucidate which plot size (row number) was sufficient to differentiate among genotypes, depending on measurement time and traits. Coefficients of correlation (r) were calculated by the Pearson product moment correlation. The used significance codes are: ‘***’ p-value < 0.001, ‘**’ p-value < 0.01 and ‘*’p-value < 0.05.

## Additional files

Additional file 1: Figure A1. Overview of the experimental field set up shown as a scheme (A), as aerial side view (B) and top down images of the three measurement arrays as used for data extraction (C). The columns shown in A represent the columns that can be seen in the field in B and C. The grey coloured plots in A are the experimental four row plots and the destructive sampling plots (numbers stand for maize genotypes), black areas are field markers and targets white areas represent edge rows, the one to three row plots or walkways. Additional file 2:
**Word document containing additional information about: 1. Experimental field setup, 2. Field management, 3. Weather information, 4. Camera set up, 5. Ground measurements, 6. Observed skewness of NDVI**_**Plant**_ **of three genotypes during the season and 7. Canopy temperature as related to air temperature and repeatability.**

## Abbreviations

NDVI: Blue band Normalized Difference vegetation Index
CC: Canopy cover
IR: Infrared
HSB: hue-saturation-brightness (color space)
TT: Thermal time
DT: Difference between canopy and air temperature
LAI: Leaf area index
LWI: Leaf weight index
IFoV: Instantaneous Field of View
AoI: Area of interest
T_C_: Canopy temperature
T_A_: Air temperature
SCO: Leaf stomatal conductance
LTMP: Leaf temperature
