# Supplementary figures and images for: Remote, aerial phenotyping of maize traits with a mobile multi-sensor approach
Source: Plant Methods. 2015 Feb 25;11:9. doi: 10.1186/s13007-015-0048-8 (PMC4365514; doi:10.1186/s13007-015-0048-8)

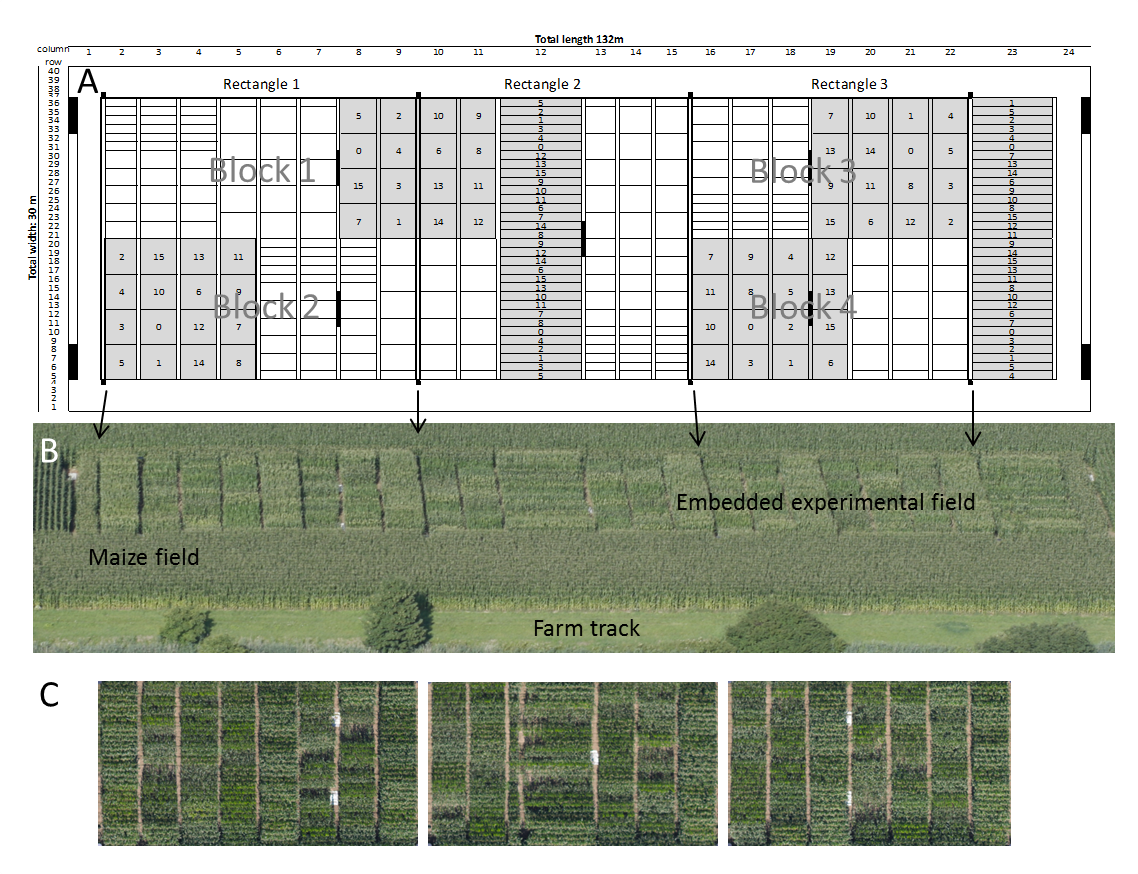

Supplement: Additional file 1: Figure A1. — Overview of the experimental field set up shown as a scheme (A), as aerial side view (B) and top down images of the three measurement arrays as used for data extraction (C). The columns shown in A represent the columns that can be seen in the field in B and C. The grey coloured plots in A are the experimental four row plots and the destructive sampling plots (numbers stand for maize genotypes), black areas are field markers and targets white areas represent edge rows, the one to three row plots or walkways. [file 13007_2015_48_MOESM1_ESM.tiff]
