# Supplementary material for: Remote, aerial phenotyping of maize traits with a mobile multi-sensor approach
Source: Plant Methods. 2015 Feb 25;11:9. doi: 10.1186/s13007-015-0048-8 (PMC4365514; doi:10.1186/s13007-015-0048-8)
Supplement: Additional file 2: — Word document containing additional information about: 1. Experimental field setup, 2. Field management, 3. Weather information, 4. Camera set up, 5. Ground measurements, 6. Observed skewness of NDVI Plant of three genotypes during the season and 7. Canopy temperature as related to air temperature and repeatability. [file 13007_2015_48_MOESM2_ESM.pdf]

Additional information

Table of Contents

1. Experimental field setup 1

2. Field management: 2

3. Weather information 3

4. Camera set up 4

5. Details on ground measurements 5

6. Examples for the observed skewness of three genotypes during the season 6

7. Thermal measurements related to repeatability 11

1. Experimental field setup

Table A1: Overview of the selected maize genotypes used in the experiment

Genotype number	Genotype	Line type	Breeder/ Company
0	Lapriora	hybrid	KWS SAAT AG, Einbeck, Germany
1	DKC2960	hybrid	DeKalb Genetics Corp., Dekalb, IL, USA
2	Tiago	hybrid	Delley seeds and plants, Delley, Switzerland (DSP)
3	Pralinia	hybrid	DSP
4	Bonfire	hybrid	DSP
5	Swiss301	hybrid	DSP
6	DSP1771E	dent line	DSP
7	DSP5009S3	dent line	DSP
8	DSP5049A31	dent line	DSP
9	DSP5145X1	dent line	DSP
10	DSP5164A3	dent line	DSP
11	DSP2563E3	flint line	DSP
12	DSP2637A	flint line	DSP
13	UH003	flint line	University of Hohenheim, Germany
14	UH008	flint line	University of Hohenheim, germany
15	SMxxx	flint line	Freiherr von Moreau Saatzucht GmbH, Altburg, Germany

2. Field management:

Preceding crop: Grain maize

Tillage operations: Plough / rotary harrow

Fertilization:

20/ April	Diammonium phosphate (DAP): 25 kg N ha^{-1}, 64 kg P ha^{-1}
04/ May	Korn-Kali®: 96 kg K ha^{-1}, 14 kg MgO ha^{-1}
10/ May	KAS 138 kg N ha^{-1}, 69 kg CaO ha^{-1}
Sum:	N: 163 kg ha^{-1}, P: 64 kg ha^{-1}, K: 96 kg ha^{-1}, MgO: 14 kg ha^{-1}

Plant protection:

27/ April	1.1 l ha^{-1} Spectrum + 2.2 l ha^{-1} Stomp Aqua
14/ July	0.125 l ha^{-1} Steward (European corn borer)

3. Weather information

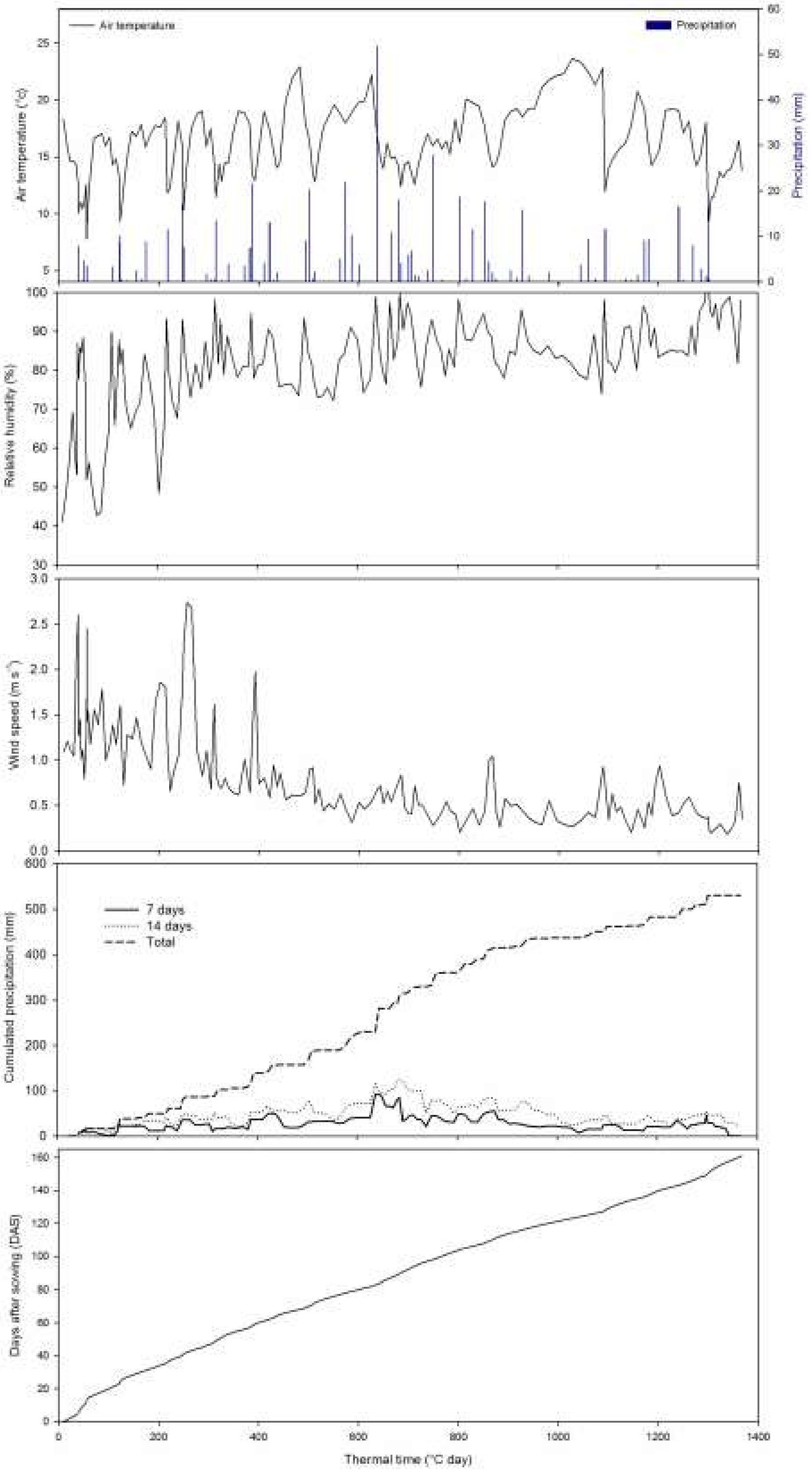

Figure A1: Cumulated precipitation, relative humidity and Wind speed during the time of the maize experiment

4. Camera set up

Table A2: Weight of the parts of the imaging tool

Part	Weight (g)	Comment
Handle frame	675 + 110+8	Aluminium + two brass screws+ 2 metal screws
Canon XSI	717	85 mm lens attached
Eos 400	770	85 mm lens attached
Connection cable and security belt	20 + 155	Cable connects the two canon cameras using the remote cable port, security belt is attached to the handling frame
VarioCam	1445	Industrial grade IR camera, needs a computer to be controlled, 75 mm lens attached
Battery+ cable	1380 + 95	Two 6 V batteries combined and attached with, IR compatible connector (self-made)
Laptop+ Accessories	1590+195+44	Laptop + Fire wire cable+ fire wire card
Sum	7204	

Table A3: Threshold settings for image segmentation for plant and soil differentiation

Flight	Date	NDVI	Grey value
1	16.06.2011	0.1	20000
2	05.07.2011	0.1	13500
3	11.07.2011	0.1	16000
4	26.07.2011	0.1	16000
5	02.08.2011	0.1	11000
6	12.08.2011	0.1	11000
7	16.08.2011	0.1	11000
8	15.09.2011	0.1	18000
9	29.09.2011	0.1	7000

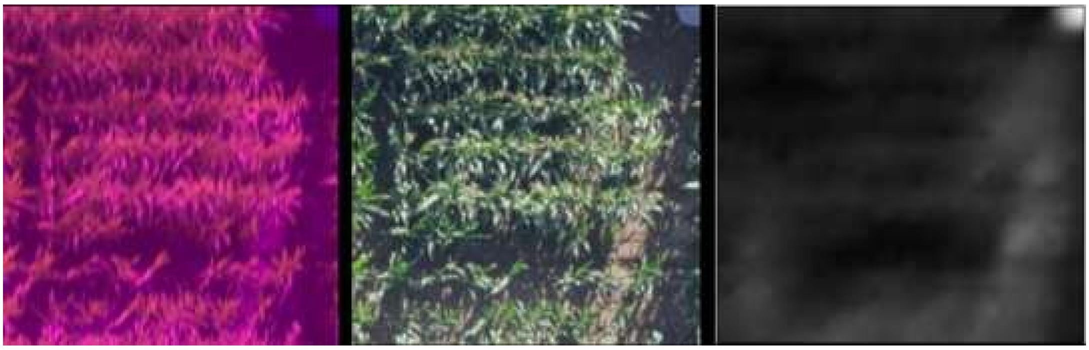

Figure A2: A sample area shown for raw NDVI, RGB and IR images (left to right) taken on 11.07.2011 (das 81). Shown is a corner position in the experimental field in rectangle 2, with a field marker in the upper right corner, tracks left and right of plots (right side downwards), and a row from top down: genotype 0, 0, 12, 12, 12, 15, 15.

5. Details on ground measurements

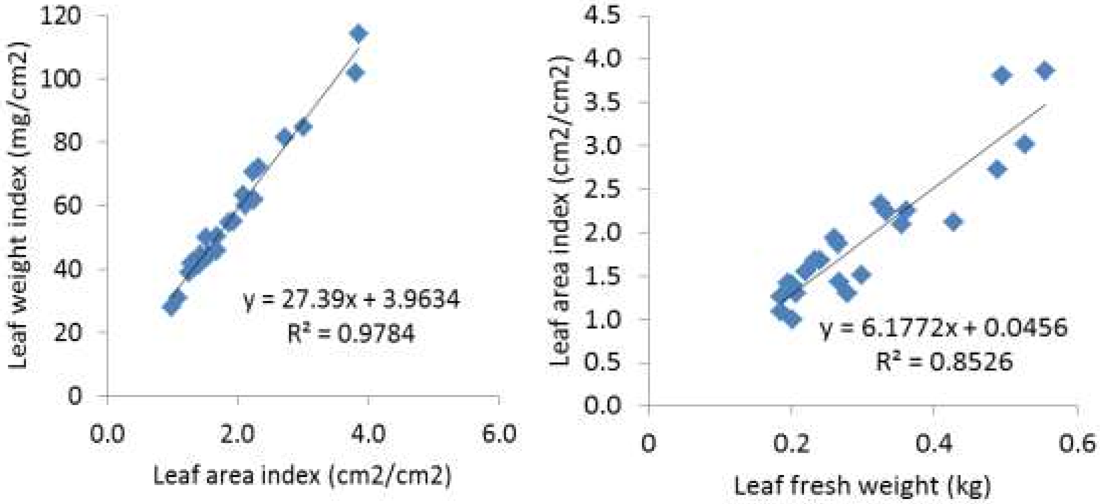

Figure A3: Tested relationship for calculation of LAI based on leaf fresh weight.

$LWI = FM_{leaf}/(SL*RD)*1000000$

LWI= leaf fresh weight index (mg cm-^{2}), FM_{leaf} = fresh matter of leaves (kg), SL = sampling length (cm), RD row distance (cm)

LAI= leaf area/(SL*RD), LAI leaf area = index

Sampled genotypes were: 3 Pralinia, 1 DKC2960, 8 DSP5049A31, 11 DSP2563E3, 13 UH003, 6 DSP1771E

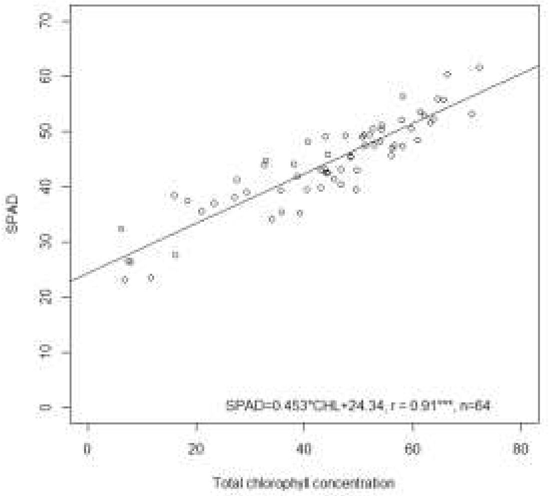

Figure A4: SPAD values as affected by chlorophyll concentration (µg cm-2) measured on ear leaves sampled at 113 DAS.

6. Examples for the observed skewness of three genotypes during the season

The skewness of the NDVI images indicates additional parameters?

The applicability of skewness as an additional parameter, quantifying the distribution of the greenness parameter NDVI (Figure A5) within the image or within the segmented area, was investigated as well. The skewness of NDVI (Figure A6) showed a different seasonal pattern than NDVI. The skewness of the $NDVI_{Plot}$ was relatively constant with a small reduction at the beginning of flowering and a stronger increase at the end of the season. Average values were usually close to zero. For $NDVI_{Plant}$ different phases were significantly more pronounced due to significant differences from normal distribution (> 0) in the early growth phase (371 °Cd) and particularly during the phase of senescence (> 940 °Cd). Repeatability of the skewness of NDVI was generally higher in larger plots (h^2 = one < two < three < four row plots) but did not always reflect the plot size differences. It strongly increased by the segmentation procedure. The repeatability of the skewness was generally lower as the one for the CC and NDVI and plot size effects were more pronounced making it less suited to differentiate among genotypes.

The NDVI skewness parameter may indicate whether an observed lower $NDVI_{Plant}$ is caused by lower overall leaf greenness or by ongoing senescence reducing the green leaf area (example in additional file 2 section 6). However, mixed pixels at the border between plant and soil may influence this signal, linking the skeweness of $NDVI_{Plant}$ also to differences in canopy cover. Indeed, the skewness of NDVI was linearly related to CC (Figure A7) where the relationship was stronger for $NDVI_{Plant}$. It can be assumed that the skewness of $NDVI_{Plant}$ mostly reflects the onset of senescence, but the mixed pixel and the senescence effect cannot be clearly disentangled here. Thus, we conclude that a pixel size of 2.5 cm on the ground (see Table 1) as in this study, is not sufficient to utilize the skewness as additional parameter.

We also tested, whether the distribution parameter the skewness of the $NDVI_{Plant}$ would be valuable as additional parameter. The skewness of $NDVI_{Plant}$ was a valuable indicator for nitrogen nutrition status in pearl millet [1]. It may be used as indicator, whether a low $NDVI_{Plant}$ is caused by a general low leaf greenness or by senescence, which increases the patchiness of green, yellow and brown leaf parts [2]. In our study, the explanatory power of skewness was limited: The increase in skewness with advanced senescence was likely related to a combination of senescent plant tissue (increased patchiness) and an increased portion of pixels containing a mixture of soil and plant signal. Thus, a high sensor resolution is a precondition to derive pure canopy pixels to measure senescence "patchiness" based on the skewness of $NDVI_{Plant}$. Because the skewness of $NDVI_{Plant}$ is linked to the amount of mixed soil and plant pixels it might serve as a parameter for the quality of the segmentation.

However, if the NDVI distribution (skewness) is of interest it turned out to be important to have three or more maize rows for a high repeatability. For the reliable detection of CC at least two or better three rows are needed.

In conclusion the 'skewness', a parameter quantifying the histogram asymmetry of NDVI signals within images, proved very useful for evaluation of segmentation quality and development of senescence.

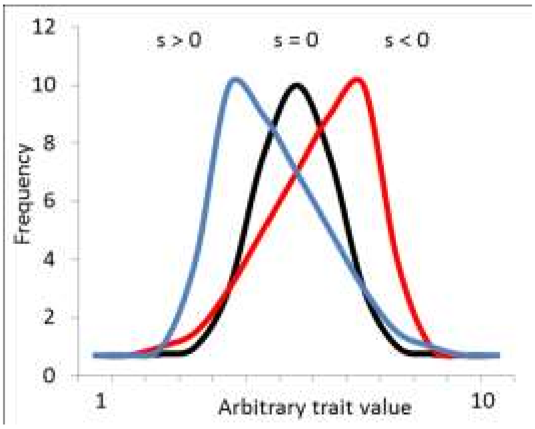

Figure A5 The skewness value (s) is a distribution parameter. A skewness value higher than zero indicates a shift towards low average NDVI values caused by a higher number of soil or senescent plant pixels. In contrast, skewness values below zero reflect a shift towards green pixels indicating a well-covered plot or plants in a fully green stage.

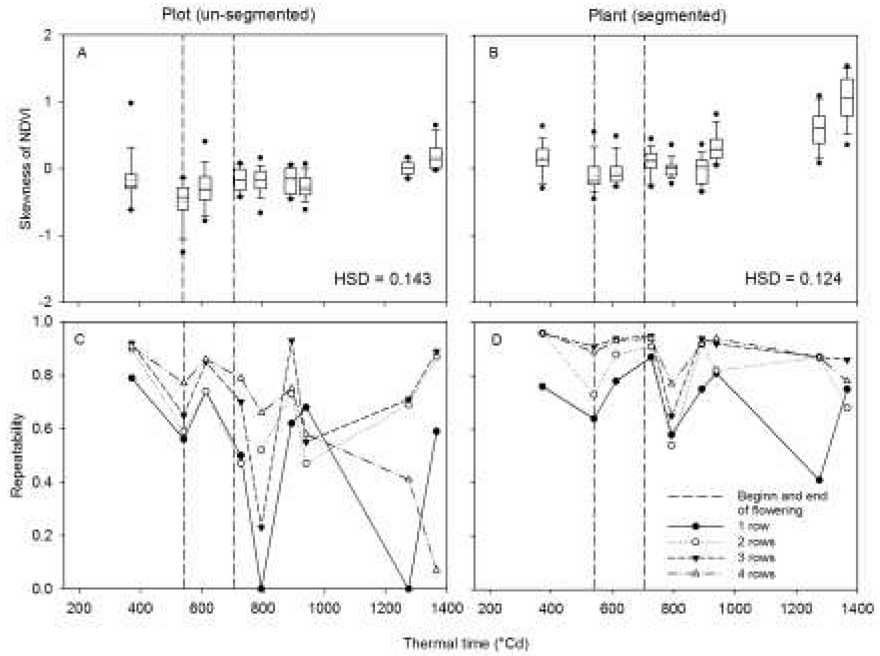

Figure A6 Skewness of NDVI and its repeatability as affected by plot size. Skewness of NDVI is shown in four row plots (A, B) and repeatability of the skewness of NDVI in one, two, three and four row plots (C, D) of plot (A, C) and plant NDVI (B, D).

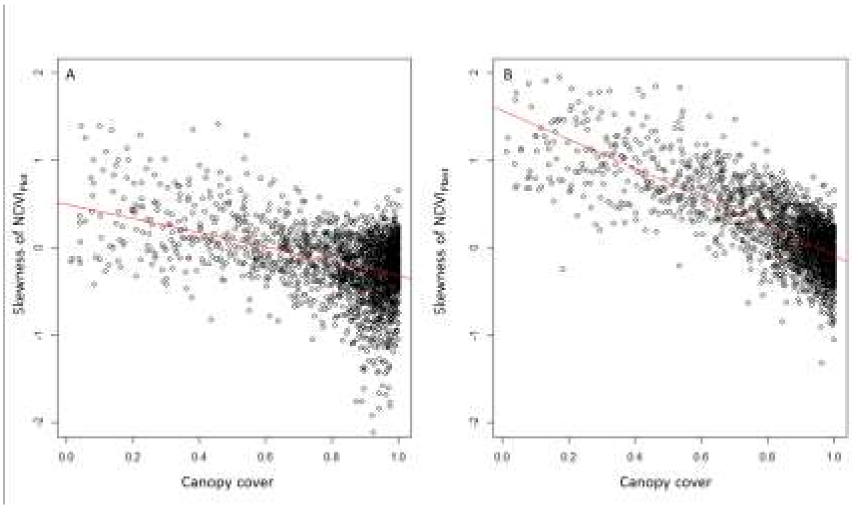

Figure A7: Relationship between canopy cover and skewness of $NDVI_{Plot}$ (A) and $NDVI_{Plant}$ (B). The indicated linear regression lines reflect the functions y = 0.5-0.82x, r2 = 0.18*** (A) and y = 1.57-1.65x, r2 = 0.56*** (B).

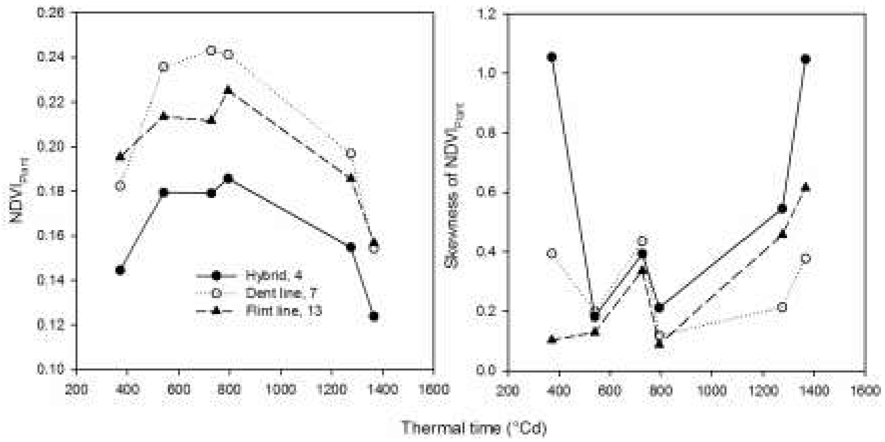

Figure A8: NDVI$_{Plant}$ and its skewness at six measurement points during the season 2011. The skewness identifies differences between genotype 7 and 13 in the late growth stages, where at the same time NDVI$_{Plant}$ doesn't.

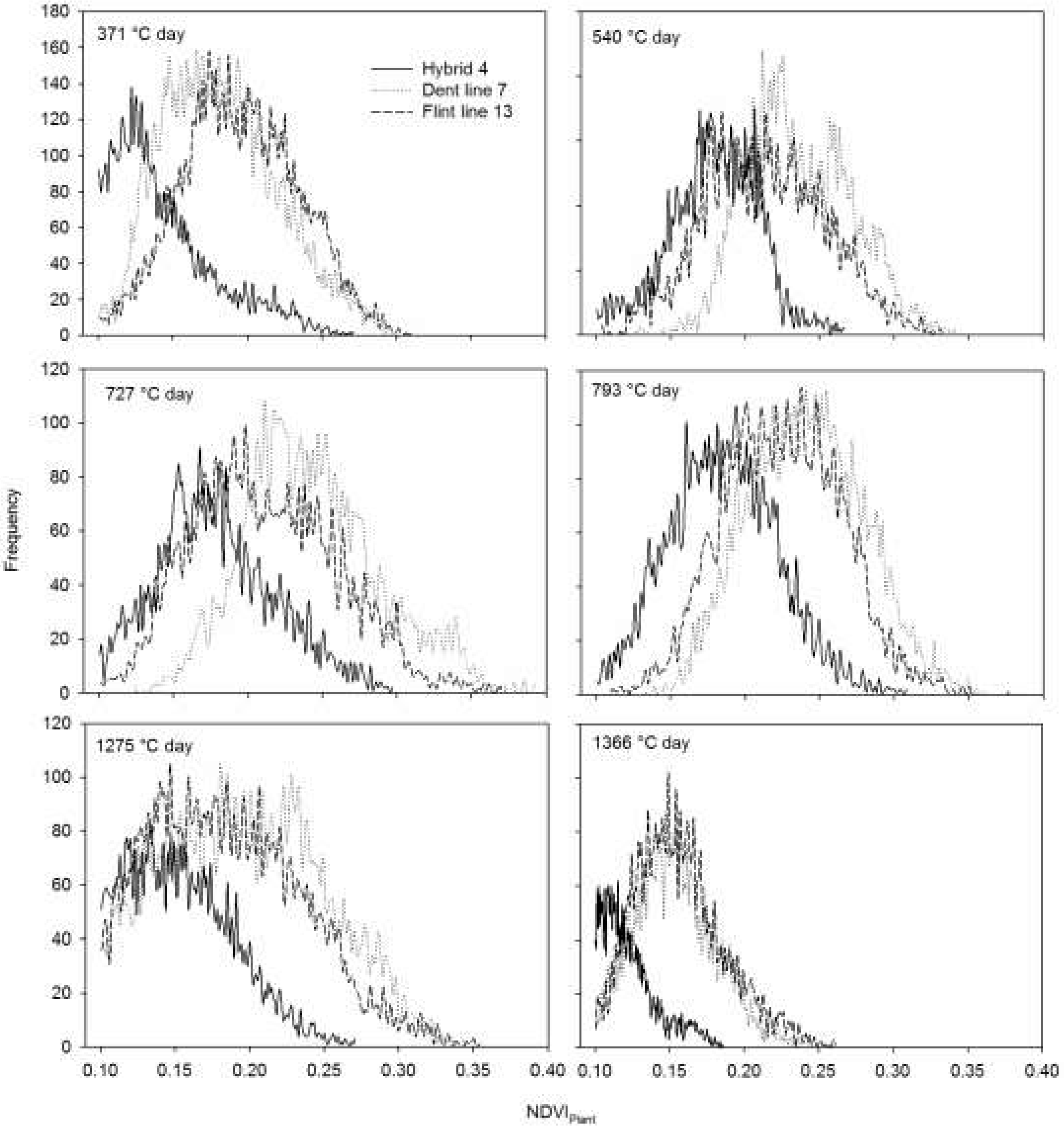

Figure A9: Distribution of NDVI$_{Plant}$ of three different genotypes shown for 6 points during seasonal development illustrating the change of the skewness.

7. Thermal measurements related to repeatability

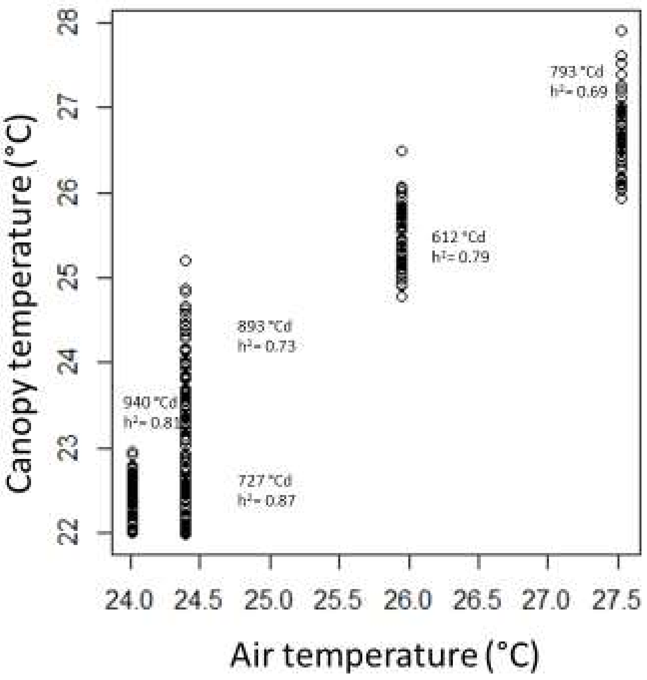

Figure A10 Canopy temperature of the three row plots (n= 64) as related to air temperature. Measurement time (°Cd) and the corresponding repeatability (h^2) is indicated.

References

1. Gerard B, Buerkert A, Hiernaux P, Marschner H: **Non-destructive measurement of plant growth and nitrogen status of pearl millet with low-altitude aerial photography (reprinted from plant nutrition for sustainable food production and environment, 1997).** *Soil Science and Plant Nutrition* 1997, **43:**993-998.
2. Thomas H: **Senescence, ageing and death of the whole plant.** *New Phytologist* 2013, **197:**696-711.
